# Robust Bayesian Fluorescence Lifetime Estimation, Decay Model Selection and Instrument Response Determination for Low-Intensity FLIM Imaging

**DOI:** 10.1371/journal.pone.0158404

**Published:** 2016-06-29

**Authors:** Mark I. Rowley, Anthonius C. C. Coolen, Borivoj Vojnovic, Paul R. Barber

**Affiliations:** 1 Institute for Mathematical and Molecular Biomedicine, King’s College London, London, United Kingdom; 2 Cancer Research UK and Medical Research Council Oxford Institute for Radiation Oncology, Department of Oncology, University of Oxford, Oxford, United Kingdom; Universidad del Pais Vasco, SPAIN

## Abstract

We present novel Bayesian methods for the analysis of exponential decay data that exploit the evidence carried by every detected decay event and enables robust extension to advanced processing. Our algorithms are presented in the context of fluorescence lifetime imaging microscopy (FLIM) and particular attention has been paid to model the time-domain system (based on time-correlated single photon counting) with unprecedented accuracy. We present estimates of decay parameters for mono- and bi-exponential systems, offering up to a factor of two improvement in accuracy compared to previous popular techniques. Results of the analysis of synthetic and experimental data are presented, and areas where the superior precision of our techniques can be exploited in Förster Resonance Energy Transfer (FRET) experiments are described. Furthermore, we demonstrate two advanced processing methods: decay model selection to choose between differing models such as mono- and bi-exponential, and the simultaneous estimation of instrument and decay parameters.

## Introduction

Optical microscopy methods are extensively and increasingly used in biomedicine. In particular, quantification of the acquired images has become essential, both in terms of morphology and in terms of intensity. More advanced fluorescence imaging techniques (e.g. confocal [1], two-photon [2], super-resolution methods [3, 4], and many others) are often used in preference to more standard methods (e.g. widefield [5]) in recent decades. Similarly, many fluorescent proteins [6] and small molecules [7, 8] are being exploited for their fluorescent properties. Advances such as these have allowed unprecedented investigations of biological cells and tissues with sub-micrometre resolution, and indeed with single molecule resolution. Many of these techniques utilise not just the intensity of fluorescence light from the sample but may also gain information from its spectrum and polarisation, as well as the probability of light emission following the excitation of the molecule (as opposed to other energy loss mechanisms). All of which can reveal details of the molecular environment, such as pH or oxygenation state [9, 10], and of molecular interactions such as those that occur between the individual proteins that enable life [11].

The finite probability of fluorescence light emission following fluorophore excitation results in a decaying profile of fluorescence intensity from a given ensemble of molecules. Since the probability per unit time is usually constant over the time period of the decay (typically nanoseconds for organic fluorescent molecules), the profile is a decaying exponential function that can be characterised by the *fluorescence lifetime* (the time to decay by 1/*e*). There are many techniques for measuring the decay profile directly in the time-domain, or indirectly, by measuring the phase shift (and loss of amplitude) of the delayed emission using modulated excitation, and subsequently determining the lifetime. A brief review of those aspects follows in this text, but it is the extraction of the fluorescence lifetime from the evidence provided by single fluorescence emission detected events, utilising Bayesian statistics in a novel way, that is the subject of this paper. Furthermore, the Bayesian framework provides a robust method for extension to more complex models of emission profile, as well the possibility of extracting additional measurements and the inclusion of prior knowledge. Because more complex models are automatically penalised by the framework if there is not sufficient data to support them, the chance of mis-interpretation is greatly reduced compared to standard techniques.

When linked with single or two-photon laser scanning techniques to form Fluorescence Lifetime Imaging Microscopy (FLIM), these techniques provide powerful tools for biological investigation. For example, molecular interactions between specific proteins in a cell can be robustly detected in living and fixed cells and tissues using FLIM by exploiting Förster Resonance Energy Transfer (FRET) [12–14]. Proteins of interest may be fluorescently labeled with specific primary antibodies and when fluorophores are chosen appropriately, FRET can be detected through a reduction in the fluorescence lifetime of one of the fluorophores. Alternatively proteins can be used directly in living cells e.g. [15]. The detection of FRET gives a clear indication that the two molecules are co-located within a distance of about 10 nm and this is the scale at which protein interactions occur [10, 16, 17].

Our previous analysis [18] is extended to offer multi-exponential decay analysis, decay model selection, and improved instrument response function (IRF) determination. The developed Bayesian analysis is based on a fully analytic system model which rigorously incorporates repetitive excitation, a multi- exponential decay model, and an improved IRF approximation in order to more accurately determine any significant limitations that may be present in a real instrument. The analysis methods presented here have advantages when photon counts are low. Under these conditions, all fitting processes have limitations, but we sought to develop a ‘gold standard’ method that can be shown to yield the best possible information from very sparse data. Our previous work [18], aimed at fitting mono-exponential decays, was shown to require about half as many detected photons to achieve decay estimates with a similar accuracy as could be obtained using other analysis methods.

**Fig 1:**
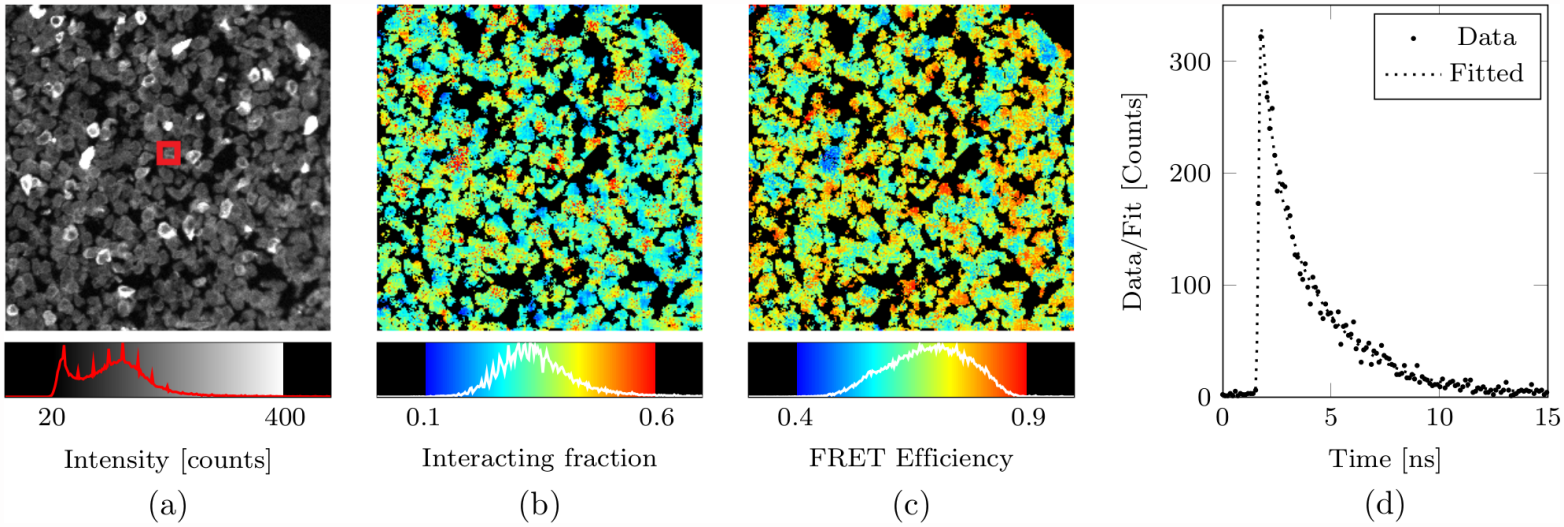
Bayesian bi-exponential analysis of cell pellet data. Lifetime analysis of FLIM data from a cell pellet sample. In (a) an intensity image having pixels with a total photon count of between about 20 and 400 were analysed (having invoked 9 × 9 spatial binning to provide sufficient photon counts for a bi-exponential analysis) using the bi-exponential Bayesian algorithm. In (b) and (c) the interacting fraction and the FRET efficiency respectively (as computed using the Bayesian estimates of the decay parameters). The size of a 9 × 9 spatial bin is indicated in (a) by a red square at the centre of the image and in (d) the decay data from from the spatial bin is shown. All of the images correspond to a 334 × 334 *μ*m field of view, and are 256 × 256 pixels. See Appendix A4 Cell Pellet Preparation for details about the sample.

As a preliminary example of what can be achieved with bi-exponential analysis in the context of a FRET experiment, Fig 1 shows the distribution of FRET efficiency and interacting fraction e.g. [19] in a cell pellet sample. A cell pellet sample is used here to demonstrate the HER2-HER3 interaction; being membrane proteins this interaction should occur fairly evenly across the sample. The separation of the two parameters, FRET efficiency and interacting fraction, is possible if bi-exponential analysis is used together with certain assumptions (see below). The simpler mono-exponential model can only reveal a single parameter, usually the *effective* FRET efficiency [19]. Many more complex FRET conditions, that are commonly ecountered, cannot be well modelled with a bi-exponential but the Bayesian framework we present lends itself well to more complex situations given sufficient data, and will provide robust and non-misleading answers. Indeed, model selection will help us to determine appropriate models in complex situations given the data acquired in conjuction with prior knowledge. The resolution of more complex models demands far greater numbers of photons; in this paper we target performance in the low intensity regime.

The data for this kind of time-domain FLIM is usually acquired using Time Correlated Single Photon Counting (TCSPC): this involves the collection of fluorescence decay photon emission times to yield a histogram of photon count (fluorescence intensity) against time, and Poisson statistics dictates that the more photons that are counted the more accurately the histogram represents the fluorescence decay. The most commonly applied analysis methods for this type of data involve the ‘direct fitting’ of a fluorescence decay model to the measured histogram. In the direct fitting approach a decay model, typically of the form

$$\begin{array}{c} I\left(t\right)=Z+\sum_{\ell =1}^{L}A_{\ell }e^{-t/\tau_{\ell }} \end{array}$$ (1)

is fitted directly to the histogram, where *I*(*t*) represents the fluorescence intensity at time *t*, *Z* represents a uniform background level, and each of the *L* decay components is described by an initial intensity *A*_*ℓ*_ and a decay lifetime *τ*_*ℓ*_. The optimal fit is determined by iteratively minimising a distance metric between the fit and the measured photon counting histogram. In performing such an analysis, the necessary convolution of *I*(*t*) with a (usually) measured instrument response function (IRF) is typically performed numerically.

The effectiveness of the ‘direct fitting’ approach depends crucially on the choice of the distance metric; different distance metrics introduce into the analysis different assumptions regarding the statistical noise in the counted photon data.

In the least-squares (LS) fitting approach, the distance metric is based on the squared difference between the measurements and the proposed fit; a choice which implicitly imposes a Gaussian ‘noise model’ that does not correspond to counting discrete events and approximates experimental reality only when the number of photons counted is large (where Gaussian statistics approximate Poisson statistics). Different adaptive and re-binning schemes have been investigated as a means of counteracting the problem of histogram bins having either zero or very low photon counts and have been found to be effective in improving the accuracy of LS estimates [20–22]. The influence of various binning ‘schemes’ was investigated, from the perspective of acquisition, by [23].

The maximum-likelihood (ML) approach to direct fitting [22, 24, 25] is based on the assumption that the noise in photon count data obeys Poisson statistics. Despite there being overwhelming consensus in the literature that ML-based direct fitting should be preferred over LS-based direct fitting, the use of LS-based fitting remains widespread in time-domain FLIM, largely down to its ease of implementation and to the fact that it is packaged with popular commercial FLIM analysis implementations. The realisation of a ML-based analysis by the simple adaption of a standard LS-based analysis using the Levenberg-Marquardt algorithm was presented by Laurence and Chromy [26].

Global analysis algorithms have been applied to time-domain FLIM [19, 27, 28] and have been demonstrated to be a powerful means by which reliable fluorescence decay parameter estimates can be obtained even in poor signal-to-noise conditions by exploiting the expected spatial invariance of some of the properties of fluorescence decays across an image (e.g. Barber *et al*. [27] assume the bi-exponential fluorescence lifetimes to be invariant across an image).

Of course, in order to obtain accurate decay parameter estimates it is necessary that the decay underlying the data be faithfully represented by the analysis model; the analysis of bi-exponential decay data with a mono-exponential model may not offer sufficient insight into the underlying fluorescence or interaction process. Similarly any significant peculiarities of the data acquisition process should also be modelled and although the influence of repetitive excitation is often acknowledged, it has only been included formally in the analysis in a small number of publications [18, 27, 29].

A number of time domain FLIM analysis methods which are not based on direct histogram fitting have also been used. A phasor analysis has been applied to time-domain FLIM [30] with an intuitive representation of the experimental data which may have a particular appeal to the non-expert. An analysis based on the Laguerre expansion has also been described e.g. [31, 32]. Although these techniques have merit in specific instances they do not offer a significant advantage when photon counts are low, are not easily extended robustly to complex situations and cannot readily include prior knowledge.

## Methods

### Modelling the photon-counting time-domain FLIM system

In time-domain FLIM, a pulsed laser is often used to periodically excite the sample, causing fluorescence emission. Fluorescence decay photons are subsequently detected by a photon-counting detector; their arrival times (relative to the pulsed excitation) being recorded electronically with a high accuracy. Over time, a set of photon arrival times that represent the fluorescence emission accumulates; it is this set that form the acquired data in our analysis. In parameterizing the system for use with this analysis, a model that captures the relevant characteristics of the system as accurately as possible, and relates a particular photon arrival time to a set of fluorescence decay and other model parameters, is developed. In the interest of developing a model that remains generally applicable, those elements of the system that exert little influence on the behaviour of the typical system (and are easily controlled experimentally through appropriate configuration of the hardware) can be safely left out. Nevertheless, it should be remembered that emitting species located at different physical sites within the microscope’s point spread function (PSF) emit with exponential decay times characteristic of each type of site, and the resulting overall decay profile is a superposition of all these emissions. When the number of sites is sufficiently large, the system can be viewed as a continuum of environments, characterized by a multi-exponential profile. In practice the photon count is far too low to be able to separate these individual components, with very similar lifetimes and the best that can be used, in the context of FLIM performed on biological material, is an approximated single exponential decay. Super resolution methods e.g. [33] may ultimately be able to improve on this by reducing the number of sites imaged in the PSF. Similarly instrumental limitations are usually determined by the type of detector. For example, photomultiplier tubes, particularly end on tubes commonly used in microscopes, exhibit wavelength-dependent transit times; detector afterpulsing can also be limiting [34] and all practical imaging systems do not respond equally to all polarisations. All these, and other effects, corrupt the acquired histogram.

**Fig 2:**
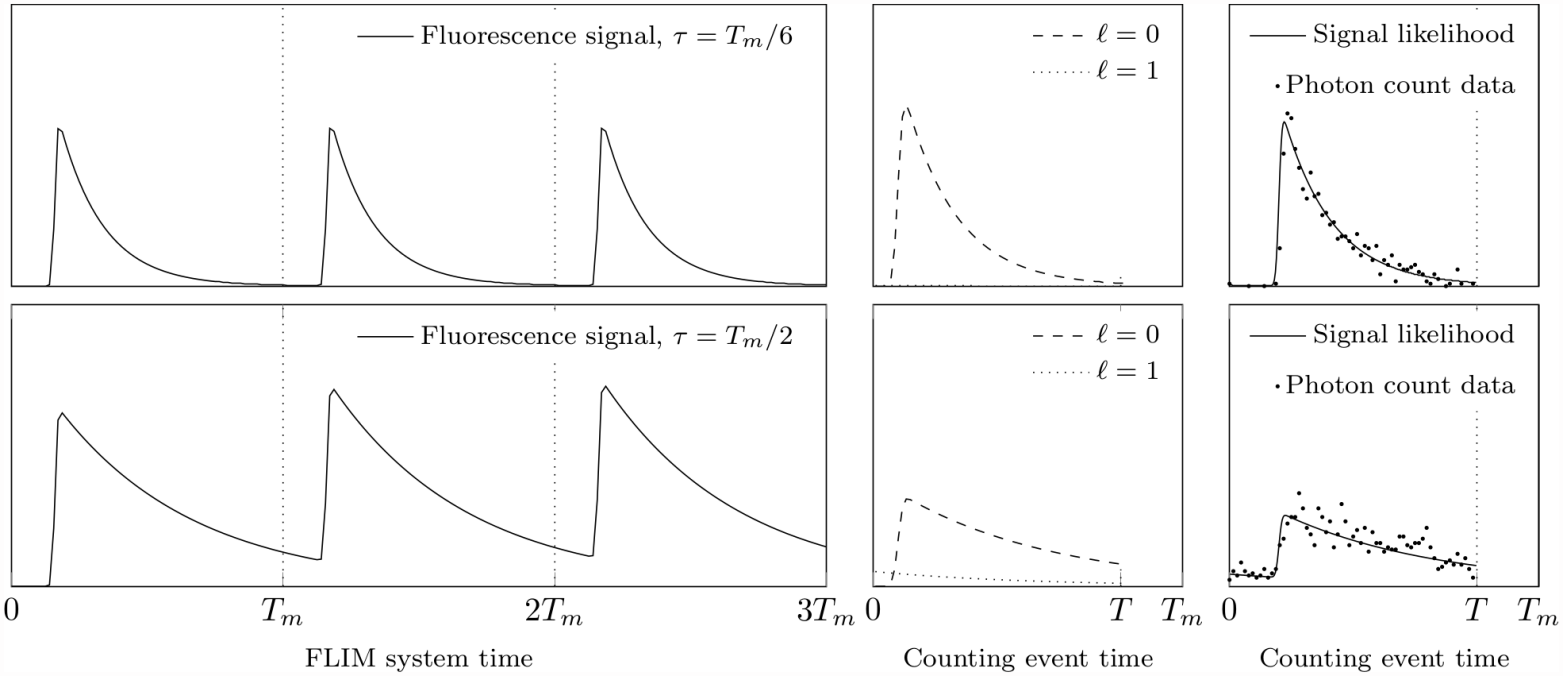
Repetitive excitation and photon counting. An illustration of the key components of the FLIM system model having repetition period *T*_*m*_ and a measurement window of duration *T*. In the upper left panel, a mono-exponential decay of lifetime *τ* = *T*_*m*_/6 as a consequence of a sample being subjected to repetitive excitation at discrete times *nT*_*m*_ (*n* = 1, 2, 3,…), the fluorescence signal being retarded by a typical IRF on progressing through the system apparatus. In the upper right panel, the normalised fluorescence signal likelihood within the measurement interval [0, *T*] (i.e. there is no likelihood of detecting a photon outside of the measurement interval (*T*, *T*_*m*_]), and typical data for such a decay having about 1,000 total photons counted into 64 bins of equal width subdividing the measurement interval. The bottom half of the figure repeats the top for a mono-exponential decay of lifetime *τ* = *T*_*m*_/2, and again shows data having about 1000 photon counts.

Considering time-resolved FLIM in fairly simple terms, as illustrated in Fig 2, a sample undergoes repetitive short-pulse excitation (repetition period *T*_*m*_) and emits fluorescence photons, some of which traverse a path through the experimental apparatus before they are detected. It is neither desirable nor likely to be advantageous to attempt to model independently the influence of all of the individual optical components of the time-domain FLIM system; instead, these effects are modelled by an overall delay *u* that the instrument imposes onto these photons. An excitation pulse of finite width and electronic jitter cause *u* to be a random variable sampled from a distribution that is described by Γ(*u*).

TCSPC involves determining the arrival time Δ*t* of a detected photon with reference to the most recent excitation pulse, and recording it as having being detected within a time interval (i.e. a bin). (In practice, *reverse-start-stop* TCSPC is used and it is actually the time between photon detection and the next excitation that is measured, it then being trivial to represent such times with reference to the preceding excitation pulse as the repetition period is known.) The following sections add details to the above description, beginning with *what goes in to the system* (i.e. repetitive excitation), followed by *what comes out of the system* (i.e. photon arrival times recorded in discrete time), and *what happens inbetween* (i.e. sample signal responds to the excitation and instrument delays the signal).

In the interest of readability, wherever possible only the key assumptions that guide the model development and significant results along the way are presented here; intermediate steps, technical and mathematical details can be found in Appendix A1 Mathematical Details.

In this analysis, the events that are analysed are photon arrival times that have been *detected* in the measurement interval [0, *T*], consequently the likelihood expressions are normalised over this interval.

**Repetitive excitation** Formalising the description above, we can write the recorded photon arrival time Δ*t* determined with respect to the most recent excitation as

$$\begin{array}{c} \Delta t=t+u-T_{m}.\left\lfloor \frac{t+u}{T_{m}}\right\rfloor \end{array}$$ (2)

where *t* is the actual *emission* time of a photon due to the fluorescence decay process and *u* captures the IRF (as described above). The final term allows for the fact that the *excitation* time may have been more than one excitation period before photon detection. The floor function is denoted by ⌊*x*⌋, such that *n* = ⌊*x*⌋ is the largest integer *n* ≤ *x*. Although detection events are correlated to the excitation ‘clock’, information about whether an event *wraps around* to subsequent detection periods is lost and defining Δ*t* in this way allows us to account for this phenomenon. A recorded arrival time Δ*t* could result from a detected photon that was due to an excitation event that occurred a period or more beforehand (*t* > *T*_*m*_); or could result from a photon emitted a number of repetition periods earlier than the most recent excitation pulse but delayed sufficiently as to be seen in the latest window (*u* > *T*_*m*_)—the likelihood of such situations depends on the decay distribution *p*(*t*) and the IRF signal distribution Γ(*u*) respectively. In reality, *p*(*t*) is much more likely to extend beyond *T*_*m*_ than is Γ(*u*).

Arrival times are recorded only during a measurement interval of duration *T* ≤ *T*_*m*_, and therefore Δ*t* ∈ [0, *T*]. The probability of arrival time Δ*t* for a photon detected in the measurement interval [0, *T*] on the periodic window, is given by

$$\begin{array}{c} p\left(\Delta t\right)=\theta \left(\Delta t\right)\theta \left(T-\Delta t\right)\left\{\frac{w_{0}}{T}+\left(1-w_{0}\right)S\left(\Delta t\right)\right\} \end{array}$$ (3)

where *S*(Δ*t*) is the likelihood of a photon from an arbitrary signal *p*(*t*) being detected at time Δ*t*, and *w*_0_ ∈ [0, 1] represents the contribution of a uniform background. The step function is denoted by *θ*(*x*), such that *θ*(*x* > 0) = 1 and *θ*(*x* ≤ 0) = 0. The probability of a signal photon having arrival time Δ*t*, while considering *all* possible emission times *t* and *all* possible delays *u*, is given by

$$\begin{array}{c} S\left(\Delta t\right)=\frac{\int_{0}^{\infty }dt\int_{0}^{\infty }du\;p\left(t\right)\Gamma \left(u\right)\delta \left(\Delta t-t-u+T_{m}.\left\lfloor \frac{t+u}{T_{m}}\right\rfloor \right)}{\int_{0}^{T}d\Delta t^{\prime }\int_{0}^{\infty }dt\int_{0}^{\infty }du\;p\left(t\right)\Gamma \left(u\right)\delta \left(\Delta t^{\prime }-t-u+T_{m}.\left\lfloor \frac{t+u}{T_{m}}\right\rfloor \right)} \end{array}$$ (4)

where the emission time *t* is distributed according to an arbitrary signal *p*(*t*) and the instrument introduces a delay *u* that is distributed according to Γ(*u*). The Dirac delta function is represented by *δ*(*x*) and exists only when *x* is equal to zero, such that ∫ *dx* *δ*(*x*) *f*(*x*) = *f*(0) for any sufficiently smooth function *f*(*x*). In transforming Eq (4) (as detailed in Appendix A1 Mathematical Details), the influence that the repetitive nature of the system has on a measured arrival time Δ*t* is instead captured in a summation, such that

$$\begin{array}{c} S\left(\Delta t\right)=\frac{1}{\Lambda (T,T_{m})}\int_{0}^{\infty }dt\;p\left(t\right)\sum_{\ell \geq 0}\Gamma \left(\ell T_{m}+\Delta t-t\right) \end{array}$$ (5)

where the normalisation constant Λ(*T*, *T*_*m*_) is given by

$$\begin{array}{c} \Lambda \left(T,T_{m}\right)=\int_{0}^{T}d\Delta t^{\prime }\;\int_{0}^{\infty }dt\;p\left(t\right)\sum_{\ell \geq 0}\Gamma \left(\ell T_{m}+\Delta t^{\prime }-t\right) \end{array}$$ (6)

Observe that Eq (5) describes the convolution of the IRF, Γ(*u*), and the pure signal *p*(*t*), as is familiar from conventional data-fitting FLIM analysis techniques. The summation has the role of accounting for the history of the signal that may be apparent in the measurement interval, such that when *ℓ* = 0 any recorded arrival time is due to a photon emitted and detected in the same measurement interval, when *ℓ* = 1 any detected photon had been emitted in the repetition period immediately preceding the measurement interval, when *ℓ* = 2 any detected photon emanated from the repetition period before that, and so on (Fig 2). Note that Eq (5) remains completely general, incorporating a uniform background proportion *w*_0_, the effects of repetitive excitation, an arbitrary instrument response Γ(*u*) and an arbitrary decay signal *p*(*t*).

**Discrete time data** Accounting for the discrete time nature of these data, the likelihood of photon arrival time Δ*t* being within a time interval (i.e. a bin) is required. Defining a bin as being an interval *b* = [*b*^L^, *b*^H^] ⊆ [0, *T*] that lies within the measurement window, and adopting the shorthand *p*(*b*) = *p*(Δ*t* ∈ *b*), the likelihood of a photon arrival time Δ*t* falling in the bin *b* is given by

$$\begin{array}{c} p\left(b\right)=\left|b\right|\frac{w_{0}}{T}+\left(1-w_{0}\right)S\left(b\right) \end{array}$$ (7)

where |*b*| = *b*^H^ − *b*^L^ denotes the width of the interval *b* = [*b*^L^, *b*^H^], and *S*(*b*) is the likelihood of a photon from the signal *p*(*t*) being detected within the bin, and is given by

$$\begin{array}{c} S\left(b\right)=\frac{1}{\Lambda (T,T_{m})}\int_{b^{L}}^{b^{H}}d\Delta t\int_{0}^{\infty }dt\;p\left(t\right)\sum_{\ell \geq 0}\Gamma \left(\ell T_{m}+\Delta t-t\right) \end{array}$$ (8)

Notice that Eqs (7) and (8) remain completely general for a system that generates discretised time-domain data due to the repetitive excitation (subject to the distributions *p*(*t*) and Γ(*u*) being normalised).

**Multi-exponential decays** The model developed so far, as given by Eqs (7) and (8), describes the photon-counting time-domain FLIM system, incorporates those effects imposed on the analysis by the design of the experimental system, that is, repetitive excitation and the collection of photon arrival times in discrete time.

A multi-exponential decay signal *p*(*t*) of the form

$$\begin{array}{c} p\left(t\right)=\theta \left(t\right)\frac{\sum_{k=1}^{K}\frac{w_{k}}{\tau_{K}}\;e^{-t/\tau_{K}}}{\sum_{k=1}^{K}w_{k}},\;\left(w,\tau \right)\in \Omega_{K} \end{array}$$ (9)

and the set Ω_*K*_ of permitted weight and lifetime values

$$\begin{array}{c} \Omega_{K}=\left\{w_{k},\tau_{K}|w_{k},\tau_{K}>0,\;\forall \;k=1,\ldots ,K,\;\sum_{k=1}^{K}w_{k}\leq 1\right\} \end{array}$$ (10)

are defined; the collection of weight and lifetime parameters of the *K* decay components being denoted by ***w*** = (*w*_1_,…,*w_K_*) and ***τ*** = (*τ*_1_,…,*τ_K_*) respectively. The introduction of Eq (9) into the model, as described by Eqs (7) and (8), subject to the requirement that $\sum_{k=1}^{K}w_{k}=1-w_{0}$, yields

$$\begin{array}{c} p\left(b\right)=\left|b\right|\frac{w_{0}}{T}+\sum_{k=1}^{K}w_{k}F\left(\tau_{k},b^{L},b^{H},I\right),\;\left(w,\tau \right)\in \Omega_{K} \end{array}$$ (11)

where *w*_*k*_ weights the contribution of an exponential decay of lifetime *τ*_*k*_ to the overall signal, the fluorescence bin-likelihood $F(\tau ,b^{L},b^{H},I)$ describing the likelihood of a photon arrival time being counted in the interval *b* = [*b*^L^, *b*^H^] due to a mono-exponential decay with lifetime *τ*, and being given by

$$\begin{array}{c} F\left(\tau ,b^{L},b^{H},I\right)=\frac{1}{\Lambda (T,T_{m})}\frac{1}{\tau }\int_{b^{L}}^{b^{H}}d\Delta t\sum_{\ell \geq 0}\int_{0}^{\infty }dt\;\Gamma \left(\ell T_{m}+\Delta t-t\right)\;e^{-t/\tau } \end{array}$$ (12)

**Analytic instrument response approximation** An approximation to the IRF comprising a weighted sum of truncated Gaussian distributions is proposed, with the aim that the asymmetry and other artifacts of a real IRF can be adequately captured. The IRF approximation is given by

$$\begin{array}{c} \Gamma \left(u,I\right)=\sum_{i=1}^{I}\gamma_{i}\frac{e^{-\frac{1}{2}{\left(u-u_{i}\right)}^{2}/\sigma_{i}^{2}}}{\sigma_{i}\sqrt{2\pi }}\frac{2\theta [u-\delta_{i}]}{1+\text{erf}(\left(u_{i}-\delta_{i}\right)/\sigma_{i}\sqrt{2})} \end{array}$$ (13)

where *γ*_*i*_ ∈ [0, 1] weights the contribution of a truncated Gaussian distribution of width *σ*_*i*_ ≥ 0, centered about a delay parameter *u*_*i*_ ≥ 0 and having a lower cut-off *δ*_*i*_ ≥ 0. The set $I=\{\gamma_{i},u_{i},\sigma_{i},\delta_{i}|i=1,\ldots ,I\}$ summarizes the parameters of the *I* instrument response components, and the weightings are subject to the constraint $\sum_{i=1}^{I}\gamma_{i}=1$. The error integral is denoted by erf(*x*) and is defined by $\text{erf}\left(x\right)=\left(2/\sqrt{\pi }\right)\int_{0}^{x}dz\;e^{-z^{2}}$. In defining this approximation the requirement for a sufficiently good configurable approximation is balanced with the desire that the necessary convolution integrals remain analytic. Approximation of the IRF by a mixture of Gaussian distributions is appropriate as any actual IRF is the convolution of many physical effects introduced by many different components of the system, including the finite width of the laser pulse, the response of the photomultiplier tube (PMT), and various effects arising from the system electronics. Repeated convolutions of any function will tend towards a Gaussian.

The introduction of Eq (13) into the fluorescence bin-likelihood model Eq (12) yields

$$\begin{array}{c} F\left(\tau ,b^{L},b^{H},I\right)=\sum_{\ell \geq 0}\frac{1}{\Lambda (T,T_{m})}\sum_{i}\tilde{\gamma_{i}}\;\Psi_{i}\left(\tau ,\ell T_{m}+b^{L},\ell T_{m}+b^{H},I\right) \end{array}$$ (14)

where the quantity $\Psi_{i}\left(\tau ,t^{L},t^{H},I\right)$ is given by

$$\begin{array}{cc} \Psi_{i}\left(\tau ,t^{L},t^{H},I\right) & =\theta \left[t^{L}-\delta_{i}\right]\left\{\chi \left(\tau ,\delta_{i},\sigma_{i},\delta_{i},u_{i}\right)-\chi \left(\tau ,t^{L},\sigma_{i},\delta_{i},u_{i}\right)\right\}\\ & \;+\theta \left[t^{H}-\delta_{i}\right]\left\{\chi \left(\tau ,t^{H},\sigma_{i},\delta_{i},u_{i}\right)-\chi \left(\tau ,\delta_{i},\sigma_{i},\delta_{i},u_{i}\right)\right\} \end{array}$$ (15)

the quantity *χ*(*τ*, *t*, *σ*, *δ*, *u*) is given by

$$\chi \left(\tau ,\;t,\;\sigma ,\;\delta ,\;u\right)=\text{erf}\left(\frac{t-u}{\sigma \sqrt{2}}\right)+e^{-\left(t-u\right)/\tau +\sigma^{2}/2\tau^{2}}\left[\text{erf}\left(\frac{\left(u-t\right)\tau +\sigma^{2}}{\sigma \tau \sqrt{2}}\right)-\text{erf}\left(\frac{\left(u-\delta \right)\tau +\sigma^{2}}{\sigma \tau \sqrt{2}}\right)\right]$$ (16)

and the quantity

$$\begin{array}{c} \tilde{\gamma_{i}}=\gamma_{i}{\left(1+\text{erf}(\left(u_{i}-\delta_{i}\right)/\sigma_{i}\sqrt{2})\right)}^{-1} \end{array}$$ (17)

is defined for compactness. The fluorescence bin-likelihood Eq (12) can be represented as the sum of contributions originating from *ℓ* ≥ 0, such that

$$\begin{array}{c} F\left(\tau ,b^{L},b^{H},I\right)=\sum_{\ell \geq 0}F_{\ell }\left(\tau ,b^{L},b^{H},I\right) \end{array}$$ (18)

where the contribution from the *ℓ*th previous excitation is given by

$$\begin{array}{c} F_{\ell }\left(\tau ,b^{L},b^{H},I\right)=\frac{1}{\Lambda (T,T_{m})}\sum_{i}\tilde{\gamma_{i}}\;\Psi_{i}\left(\tau ,\ell T_{m}+b^{L},\ell T_{m}+b^{H},I\right) \end{array}$$ (19)

It is worthy of note that, although the model incorporates rigorously any history of the signal that may be present in the recorded arrival time data, in practice, for decay lifetimes considerably shorter than the repetition period, the summation over *ℓ* need only include the first two terms. In the case that a decay lifetime is not significantly smaller than the repetition period, the summation should include more terms as appropriate.

### Bayesian analysis of the system model

The Bayesian framework is now applied to the system model developed above with the purpose of quantifying probabilistically the model parameters of some fluorescence decay data and also to provide answers to the often unasked questions regarding the nature of both the fluorescence decay and of the FLIM instrument. In particular, the following are explored:

- **Parameter estimation**
  What is the probability of a set of fluorescence decay model parameter values given the detected data, the decay model, and the parameterized instrument response approximation?
- **Model selection**
  How many exponential decay components, *K*, are likely to have yielded the decay data?
- **Instrument response determination**
  What is the most likely form of the IRF given the *fluorescence decay* data?

The following notation is introduced and shall be used throughout the remainder of this document:

- **The data**
  The data *D* = {(*b*_*j*_, *c*_*j*_)|*j* = 1,…,*M*} comprise a set of *M* bin-count pairs (*b*_*j*_, *c*_*j*_) where *b*_*j*_ represents the *j*th bin (i.e. interval $b_{j}=\left[b_{j}^{L},b_{j}^{H}\right]\in \left[0,T\right]$) and *c*_*j*_ is the number of photons recorded as having been counted into that bin. It is required that none of the bins overlap (i.e. *b*_*j*_ ∩ *b*_*k*_ = ∅, ∀*j* ≠ *k*) and that their union forms the measurement interval (i.e. $\cup_{j=1}^{M}b_{j}=\left[0,T\right]$).
- **The decay model**
  The fluorescence decay model is denoted by $H_{K}$, where *K* exponential decays of lifetime *τ*_*k*_ contribute to the overall decay according to weight *w*_*k*_ as defined by Eq (9). The notation ***w**_*K*_* = (*w*_1_,…,*w*_*K*_) and ***τ**_*K*_* = (*τ*_1_,…,*τ*_*K*_) shall also be used.
- **The parameterized IRF approximation**
  The characteristics of the FLIM equipment are denoted by $I=\{\gamma_{i},u_{i},\sigma_{i},\delta_{i}|i=1,\ldots ,I\}$, which defines an instrument response approximation composed of *I* weighted, truncated Gaussian distributions, as defined by Eq (13).

The Bayesian analysis developed in this section intentionally does not explicitly incorporate any particular form for the required prior distributions, in order that the expressions developed remain general and can be used as a starting point and be suitably updated on making specific the choice of prior distribution.

**Decay parameter estimation** Most commonly, it is the fluorescence decay parameter estimates that are of greatest interest to the experimentalist, with both the IRF approximation, I, and the decay model, $H_{K}$, having been either determined or assumed. The posterior distribution gives the likelihood of fluorescence decay parameter values, (***w**_K_*, ***τ**_K_*), given the data, *D*, the IRF approximation, I, and the decay model, $H_{K}$, and its hyperparameter(s), ***α**_K_*, and is given by

$$\begin{array}{cc} p(w_{K},\tau_{K}|D,H_{K},\alpha_{K},I) & =\frac{1}{Z_{K}}p\left(w_{K},\tau_{K}|H_{K},\alpha_{K}\right)p\left(D|w_{K},\tau_{K},H_{K},I\right)\\ & =\frac{1}{Z_{K}}p\left(w_{K},\tau_{K}|H_{K},\alpha_{K}\right)\prod_{j=1}^{M}p{\left(b_{j}|w_{K},\tau_{K},H_{K},I\right)}^{c_{j}} \end{array}$$ (20)

where the normalisation constant, $Z_{K}=Z_{K}({ℋ}_{K},\;\alpha_{K},D,ℐ)$, is given by

$$\begin{array}{c} Z_{K}=\int dw_{K}\;d\tau_{K}\;p\left(w_{K},\tau_{K}|H_{K},\alpha_{K}\right)\prod_{j=1}^{M}p{\left(b_{j}|w_{K},\tau_{K},H_{K},I\right)}^{c_{j}} \end{array}$$ (21)

and the shorthand $\int dw_{K}d\tau_{K}=\int \ldots \int dw_{1}d\tau_{1}\ldots dw_{K}d\tau_{K}$ has been introduced for compactness.

Of course, as any parameter estimates, (***w**_K_*, ***τ**_K_*), derived from Eq (20) are conditioned not only on the data, *D*, but also on the decay model, $H_{K}$, (and its hyperparameter(s), ***α**_K_*), and instrument response characterization, I, it should be expected that they be most reasonable when the appropriate $H_{K}$ and I have been employed throughout the analysis.

**Decay model selection** Here, in order to address such questions as “*Should mono-exponential or bi-exponential analysis be used?*”, the Bayesian framework is employed to determine the most applicable *fluorescence decay* model $H_{K}$ given the data, where it is assumed in this analysis that the IRF parametrization I has been appropriately determined. In this work, the most probable decay model and the most probable hyperparameter(s) are determined together, their posterior distribution offering a quantitative measure of the likelihood of the recorded data being due to the model $H_{K}$ and its hyperparameter(s) *α_K_*, as given by

$$\begin{array}{c} p\left(H_{K},\alpha_{K}|D,I\right)=\frac{p\left(H_{K},\alpha_{K}\right)Z_{K}\left(H_{K},\alpha_{K},D,I\right)}{\sum_{H_{K}^{\prime }}\int d\alpha_{K^{\prime }}\;p\left(H_{K^{\prime }},\alpha_{K^{\prime }}\right)Z_{K^{\prime }}\left(H_{K^{\prime }},\alpha_{K^{\prime }},D,I\right)} \end{array}$$ (22)

where $p({ℋ}_{K},\;\alpha_{K})$ is the prior probability of decay model $H_{K}$ and its hyperparameter(s) *α_K_*, and in the denominator the summation is over all candidate decay models. The relative likelihood of candidate decay models, for example models $H_{K}$ and $H_{K}^{\prime }$, can be obtained by the pairwise comparison of the evidence for those models, that is

$$\begin{array}{cc} & \frac{p(H_{K},\alpha_{K}|D,I)}{p(H_{K^{\prime }},\alpha_{K^{\prime }}|D,I)}=\frac{p(H_{K},\alpha_{K})}{p(H_{K^{\prime }},\alpha_{K^{\prime }})}\frac{Z_{K}\left(H_{K},\alpha_{K},D,I\right)}{Z_{K^{\prime }}\left(H_{K^{\prime }},\alpha_{K^{\prime }},D,I\right)} \end{array}$$ (23)

Alternatively, the most probable decay model and its hyperparameters conditioned on the data can be sought by finding the maximum of Eq (22). On writing $p({ℋ}_{K},\;\alpha_{K})=p({ℋ}_{K})p(\alpha_{K}|{ℋ}_{K})$, the most probable decay model $H_{K}^{⋆}$ and its optimal hyperparameters $\alpha_{K}^{⋆}$ are given by

$$\begin{array}{cc} \left(H_{K}^{⋆},\alpha_{K}^{⋆}\right) & =\underset{H_{K},\alpha_{K}}{\mathrm{argmax}}\left[p\left(H_{K}\right)p\left(\alpha_{K}|H_{K}\right)Z_{K}\left(H_{K},\alpha_{K},D,I\right)\right]\\ & \;\;\;\;=\underset{H_{K}}{\mathrm{argmax}}\left[p\left(H_{K}\right)\max_{\alpha_{K}}\left[p\left(\alpha_{K}|H_{K}\right)Z_{K}\left(H_{K},\alpha_{K},D,I\right)\right]\right]\\ & =\underset{H_{K}}{\mathrm{argmax}}\left[p\left(H_{K}\right)p\left(\alpha_{K}^{⋆}|H_{K}\right)Z_{K}\left(H_{K},\alpha_{K}^{⋆},D,I\right)\right] \end{array}$$ (24)

where the quantity $Z_{K}({ℋ}_{K},\;\alpha_{K},D,ℐ)$ is as defined by Eq (21), the optimal hyperparameter(s) are given by

$$\begin{array}{cc} \alpha_{K}^{⋆} & =\underset{\alpha_{K}}{\mathrm{argmax}}\left[p\left(\alpha_{K}|H_{K}\right)Z\left(H_{K},\alpha_{K},D,I\right)\right] \end{array}$$ (25)

and $p(\alpha_{K}|\;{ℋ}_{K})$ is the prior distribution (the so-called hyperprior) of the hyperparameter(s) ***α**_K_* for the decay model $H_{K}$.

**Instrument response determination** The measurement of an instrument response can sometimes be a difficult practical problem or the IRF may vary over the image in some unpredictable way. These situations are more likely to occur in fields other than microscopy, where, for example, images of natural scenes may be obtained with a fluorescence lifetime camera. This section develops the Bayesian determination of the instrument response approximation parameters from the fluorescence decay data itself. Denoting by $I=\{u_{i},\sigma_{i},\delta_{i}|i=1,\ldots ,I\}$ the parameter values of the instrument response approximation Γ(*u*) (Eq (13)), the posterior distribution of the instrument response parameter values is given by

$$\begin{array}{cc} & p\left(I|D\right)=\frac{p\left(I\right)\sum_{H_{K}}p\left(H_{K}\right)Z_{K}\left(H_{K},\alpha_{K},D,I\right)}{\int dI^{\prime }\;p\left(I^{\prime }\right)\sum_{H_{K}}p\left(H_{K}\right)Z_{K}\left(H_{K},\alpha_{K},D,I\right)} \end{array}$$ (26)

Of course, should the data be acquired using a fluorophore known to exhibit a purely *K*-exponential decay, the above simplifies to yield

$$\begin{array}{c} p\left(I|D,H_{K}\right)=\frac{p\left(I\right)Z_{K}\left(H_{K},\alpha_{K},D,I\right)}{\int dI^{\prime }\;p\left(I^{\prime }\right)Z_{K}\left(H_{K},\alpha_{K},D,I\right)} \end{array}$$ (27)

Additionally, it is also possible to estimate the instrument response parameters at the same time as estimating the fluorescence decay parameters. Again, under the assumption of a purely *K*-exponential decay, the posterior to be calculated simplifies yet further to give

$$\begin{array}{c} p\left(w_{K},\tau_{K},I|D,H_{K}\right)=\frac{p\left(I\right)p\left(w_{K},\tau_{K}\right)p\left(D|H_{K},\alpha_{K},D,I,w_{K},\tau_{K}\right)}{\int dI^{\prime }\;p\left(I^{\prime }\right)Z_{K}\left(H_{K},\alpha_{K},D,I\right)} \end{array}$$ (28)

### Software Implementation

The Bayesian analysis algorithms were implemented in the C programming language and incorporated into the TRI2 (Time Resolved Imaging 2) image processing software package [19, 27].

The optimal mono- and bi-exponential parameter estimates are those which maximise the posterior distribution as given by Eq (20), or equivalently, those which minimise the quantity $-\text{ln}(p(w_{K},\tau_{K}|D,{ℋ}_{K},\alpha_{K},ℐ))$. In our implementation these values are determined by locating the global minimum using two different approaches; one in which the global minimum is located in the “continuous” parameter space by means of the downhill simplex or simulated annealing algorithms [35], and another in which an exhaustive search of the discretised parameter space is performed, thereby enabling faster determination of the optimal values and easier viewing of the posterior and marginal distributions.

A Gaussian approximation, as developed in e.g. [36], to the mono- and bi-exponential parameters posterior distributions Eq (20) was employed for the implementation of the Bayesian model selection algorithm. In adopting such an approach the “model evidence”, as given by Eq (21), may be approximated using only the optimal parameter values and the (first and second) derivatives of the quantity *S*(***w**_K_*, ***τ**_K_*) = ‒ln[*p*(***w**_K_*, ***τ**_K_*)*p*(*D*|***w**_K_*, ***τ**_K_*)], which in this work were determined analytically. The primary advantage of using this approach is that the requirement for numerical integration to determine the “model evidence” is avoided.

Determination of the optimal IRF approximation has been implemented using a simulated annealing algorithm [35] and under the assumption that the fluorescence decay is known to be either mono- or bi-exponential. The inclusion of too many Gaussian components in the IRF approximation Eq (13) and the consequences of over-fitting could be avoided by the development of a Bayesian IRF approximation model selection algorithm to determine the optimal number of Gaussian components and their parameters; this has not been investigated to date though would be an achievable extension to the current implementation should it be deemed to offer sufficient advantage to the analysis.

## Results

The performance of the Bayesian algorithms was compared to that of the direct-fitting approach using ML and LS for the analysis of synthetic data simulating various mono- and bi-exponential decay conditions and a typical TCSPC system (see Appendix A2 Synthetic Data). The performance of the different methods was also compared for the analysis of human carcinoma cell and human breast cancer tissue experimental data (see Appendices A3 Human Epithelial Carcinoma Cell Preparation and A5 Human Breast Cancer Tissue Preparation).

The Bayesian mono- and bi-exponential decay analysis algorithms were tested against ML and LS, all approaches operating directly on the accumulated histogram. The ML estimation routines were implemented as described in [26], and are based on the modified Levenberg-Marquardt (MLM) algorithm. The LS implementation is also based upon the MLM algorithm and is described in [19, 27]. As the repetitive nature of TCSPC excitation is not accounted for in any of the established analysis techniques used for comparison, in order to avoid their potential effects the ML and LS analysis routines consider only a window of the collected data that excludes time points before the rise of the transient has occurred. Automated selection of the valid data window is discussed in [27]. The Bayesian analysis routines operate on all of the available data, except for small portions at the start and end of the transient, potentially corrupted by consequences of dithering applied to time measurements of the time-amplitude converter in the TCSPC electronics [34].

The Bayesian-determined optimal single Gaussian IRF approximation, having a FWHM width of 0.129 ns (i.e. a standard deviation of 0.055 ns) centered about a delay of 2.067 ns, was determined from a single high-count data IRF measurement (about 5 million photon counts) and was used for the analysis of synthetic data presented below.

### Mono-exponential parameter estimation

The mono-exponential parameter estimates obtained with this model are in close agreement with those reported in [18] using a simpler model. In particular, when applied to low count synthetic data (having a background component of about 10%), our Bayesian algorithms enable a decrease of about a factor of two in the number of photon counts required, and therefore in the imaging duration, for mono-exponential lifetime estimation with an accuracy of about 10%. Similarly, when applied to experimental data, Bayesian mono-exponential analysis of the data from imaging human epithelial carcinoma cells stably expressing cdc42-GFP again yielded a tighter lifetime distribution than those obtained using ML and LS methods. This indicates a reduction in the spread of the random error of the *analysis* and results that are more representative of the *biological* distribution of lifetime values due to local variations in the cellular environment.

The enhanced model described in this paper offers improvements over the model of [18] in the case of decays having a very slow decay component, and in situations where the IRF is not well approximated by a single Gaussian.

### Bi-exponential parameter estimation

In bi-exponential decay analysis, the lifetimes, *τ*_1_ and *τ*_2_, and initial amplitudes, *A*_1_ and *A*_2_, of the two decay components are estimated from the fluorescence decay data.

**Fig 3:**
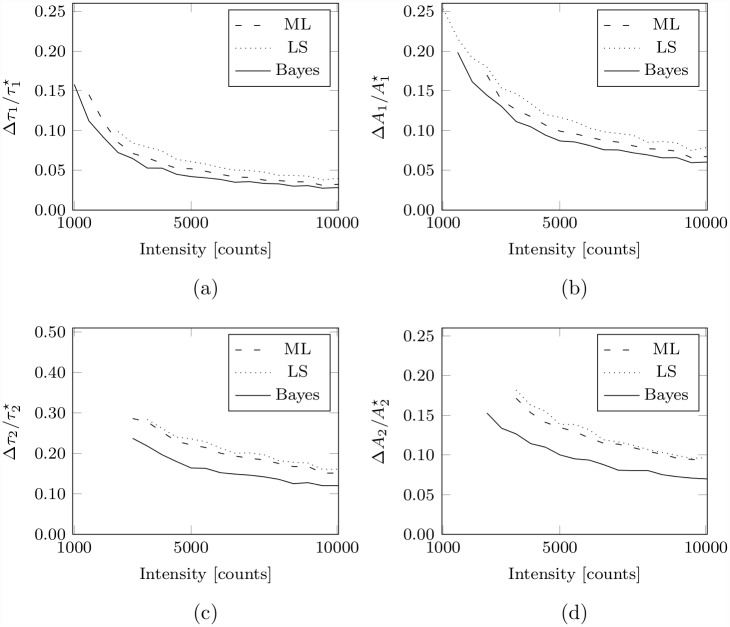
Bi-exponential parameter estimation at low photon counts. The uncertainty, as measured by the standard deviation of the estimated parameter distribution, in the bi-exponential decay parameter estimates obtained using ML, LS, and Bayesian analysis for the analysis of synthetic data simulating a bi-exponential decay ($\tau_{1}^{⋆}=2.0\;\text{ns}$, $\tau_{2}^{⋆}=0.5\;\text{ns}$, $A_{1}^{⋆}=A_{2}^{⋆}$) for a range of intensities between 10^3^ and 10^4^ photon counts. In (a) and (c) the fractional errors in the estimated decay lifetimes versus photon count; in (b) and (d) the fractional errors in the initial amplitudes of the two decay components. In all cases, the normalised width is displayed only when the respective estimates are not biased by more than 5% of the true value.

A comparison of the performance of the Bayesian bi-exponential algorithm with LS and ML is shown in Fig 3 for the analysis of synthetic data simulating bi-exponential decays at low photon counts. The fractional errors in the estimated lifetimes and initial amplitudes versus photon count are shown in (Fig 3a and 3c) and (Fig 3b and 3d) respectively for simulated bi-exponential decays having lifetimes $\tau_{1}^{⋆}=2.0\;\text{ns}$ and $\tau_{2}^{⋆}=0.5\;\text{ns}$, and having equal initial amplitudes i.e. $A_{1}^{⋆}=A_{2}^{⋆}$.

The Bayesian bi-exponential algorithm provides parameter estimates to a greater precision, as measured by the standard deviation of the estimated parameter distributions, than is achieved using ML or LS analysis, as is evident on visual inspection of Fig 3; the Bayesian algorithm offers superior estimates for all of the bi-exponential decay parameters for all intensities between about 10^3^ and 10^4^ photon counts. At an intensity of about 5×10^3^ photon counts, the lifetime of the slower decay component, *τ*_1_, is estimated to a precision of about 4.2% by our Bayesian algorithm; at the same intensity ML and LS achieve a precision of about 5.2% and 6.1% respectively. Indeed, to achieve the precision that is offered by the Bayesian algorithm at 5×10^3^ photon counts, ML requires an intensity of almost 6.5×10^3^ photon counts and LS requires about 9×10^3^ photon counts. A similar improvement in precision is achieved for the estimates of the corresponding initial amplitude, *A*_1_, at 5×10^3^ photon counts; the Bayesian algorithm offers estimates within a precision of 8.9%, whereas ML and LS achieve a precision of 10.3% and 11.7% respectively. The precision of the estimated lifetime and initial amplitude of the faster decay component is expected to be inferior to that of the slower component, as for such a decay the slower component contributes roughly four times as many of the counted photons to the intensity than the slower decay component. However, it is for the characterisation of the faster decay component that the Bayesian algorithm provides greatest advantage over ML and LS. The faster decay lifetime, *τ*_2_, is estimated to a precision of 16.4% by the Bayesian algorithm at 5×10^3^ photon counts; ML and LS do not offer such precision below intensities of about 8.5×10^3^ and 9.5×10^3^ photon counts respectively. The initial amplitude of the faster decay component, *A*_2_, is also estimated with greater precision by the Bayesian algorithm; at 5×10^3^ photon counts the Bayesian algorithm achieves a precision of 10.0%, as compared to a precision of 13.5% offered by ML and 13.8% offered by LS analysis.

In a biological example where levels of protein-protein interactions can be determined by FRET (via FLIM), we can demonstrate the use of the bi-exponential model. We are thus making the assumptions that the donor has a single dominant decay path, resulting in mono-exponential kinetics, and that the target proteins (for example, HER2 and HER3 for the cell pellet data (Appendix A4 Cell Pellet Preparation) illustrated in Fig 1) readily form a stable dimer where the donor to acceptor distance is relatively stable and consistent. Thus bi-exponential analysis is appropriate to resolve the interacting (dimerised) and non-interacting populations. In such situations, the FRET efficiency and interacting fraction are biologically relevant quantities that can then be determined from the estimated decay lifetimes *τ*_1_ and *τ*_2_ and the initial amplitudes *A*_1_ and *A*_2_ respectively, as follows,

- FRET efficiency: *E* = 1 − *τ*_2_/*τ*_1_, *τ*_1_ > *τ*_2_,
- Interacting fraction: *F*_2_ = *A*_2_/(*A*_1_ + *A*_2_).

The FRET efficiency *E* can be used to quantify the separation of fluorophore pairs and the interacting fraction *F*_2_ provides a measure of the proportion of molecules undergoing FRET.

Of course, in such experiments, superior bi-exponential parameter estimates lead to more precise estimates of derived quantities such as the FRET efficiency and interacting fraction. Indeed, the FRET efficiency for the bi-exponential decay data analysed for Fig 3 was estimated to a precision of 4.5% using the Bayesian bi-exponential lifetime estimates at an intensity of 5×10^3^ photon counts; the ML and LS estimates resulted in a precision of 6.2% and 6.3% respectively. Similarly, a precision of 8.2% was achieved for the interacting fraction estimated from the Bayesian bi-exponential initial amplitudes; ML and LS offered a precision of 9.5% and 10.3% respectively.

**Fig 4:**
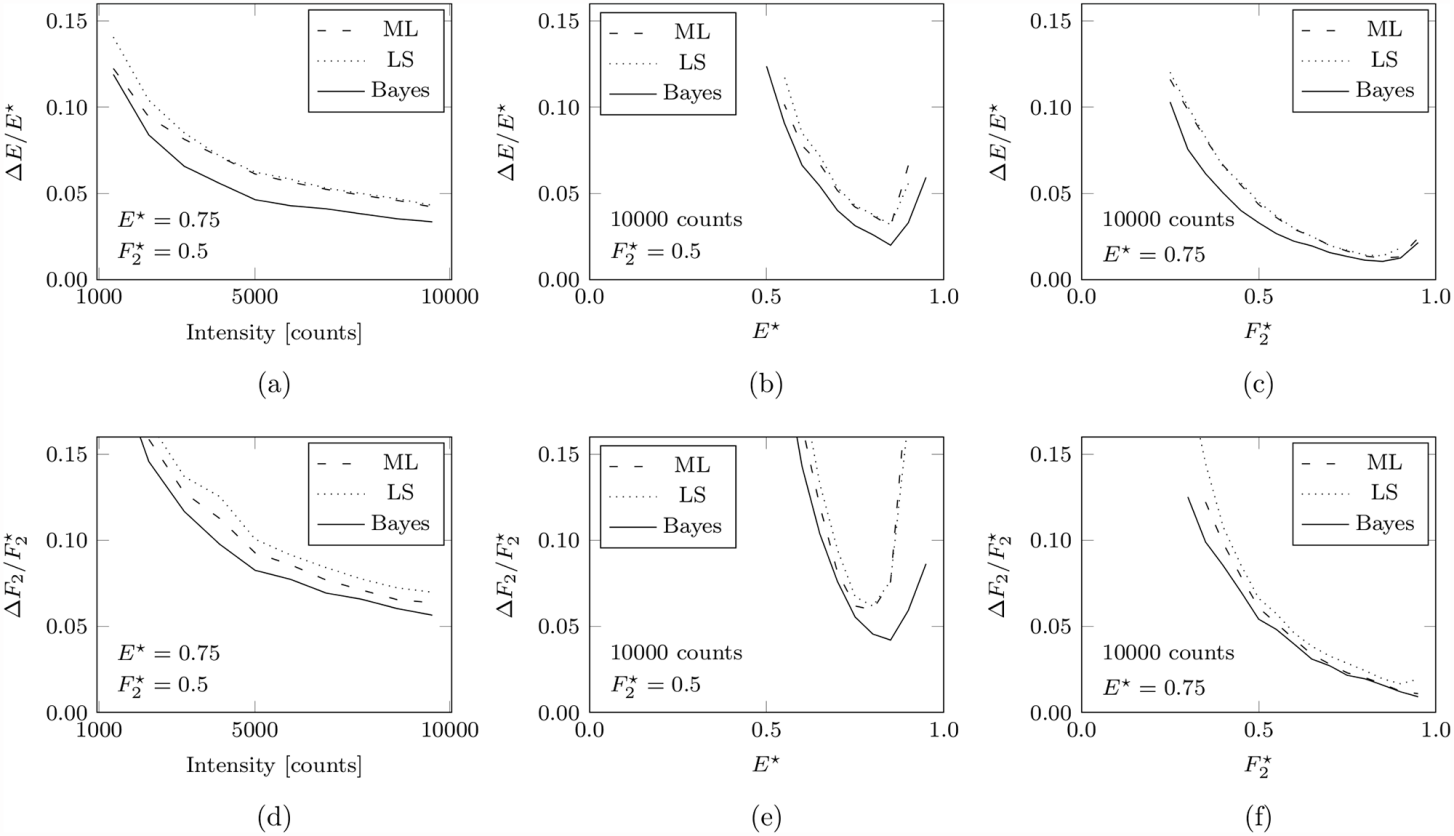
FRET efficiency and interacting fraction estimation at low photon counts. The uncertainty, as measured by the standard deviation of the estimated parameter distribution, in the bi-exponential decay parameter estimates obtained using ML, LS, and Bayesian analysis for the analysis of synthetic data simulating a bi-exponential decay. In (a) and (d) are the fractional errors in FRET efficiency and interacting fraction respectively versus photon count, in (b) and (e) the same for different FRET efficiencies *E*^⋆^, and in (c) and (f) for different interacting fractions $F_{2}^{⋆}$. In all cases, the normalised width is displayed only when the respective estimates are not biased by more than 5% of the true value. See main text for details.

A comparison of the performance of the Bayesian bi-exponential algorithm with LS and ML is shown in Fig 4 for the analysis of synthetic data simulating three FRET efficiency and interacting fraction conditions at low photon counts. The fractional errors in FRET efficiency and interacting fraction versus photon count are shown in (Fig 4a and 4d), the same errors are shown in (Fig 4b and 4e) and (Fig 4c and 4f) for different FRET efficiencies and for different interacting fractions respectively.

The fractional errors in FRET efficiency and interacting fraction versus photon count (Fig 4a and 4d) are plotted for synthetic data simulating a bi-exponential decay having a constant FRET efficiency of *E*^⋆^ = 0.75 (i.e. lifetimes of $\tau_{1}^{⋆}=2.0\;\text{ns}$ and $\tau_{2}^{⋆}=0.5\;\text{ns}$) and an interacting fraction of $F_{2}^{⋆}=0.5$ (i.e. $A_{1}^{⋆}=A_{2}^{⋆}$), for intensities between about 10^3^ and 10^4^ photon counts. The improvement in the estimated FRET efficiency offered by Bayesian analysis is a consequence of superior lifetime estimates compared to those from ML and LS analysis. At an intensity of about 5×10^3^ photon counts the FRET efficiency derived from Bayesian lifetime estimates has an uncertainty of about 4.7%, whereas the estimates due to ML and LS analysis have an uncertainty of about 6.2% and 6.4% respectively. Bayesian analysis also offers improved estimates of the interacting fraction; at an intensity of about 5×10^3^ photon counts the interacting fraction determined from the Bayesian bi-exponential parameter estimates has an uncertainty of about 8.3% as compared to about 9.6% and 10.3% for ML and LS respectively.

The performance for different FRET efficiencies is shown in (Fig 4b and 4e) for synthetic data having an interacting fraction of $F_{2}^{⋆}=0.5$ (i.e. $A_{1}^{⋆}=A_{2}^{⋆}$) and an intensity of about 10^4^ photon counts. Bayesian analysis provides a slight improvement in the estimation of the FRET efficiency and the interacting fraction and could therefore offer more precise estimates at a given FRET efficiency or estimates within a given precision for an increased range of FRET efficiencies. However, it is evident that at such an intensity the uncertainty in the estimated FRET efficiency exceeds 10% and increases rapidly for all of the analysis techniques for actual FRET efficiencies of less than about 0.55 and that an equally rapid deterioration in the estimated interacting fraction occurs at FRET efficiencies lower than about 0.65. The estimated interacting fraction demonstrates a bias of greater than 5% for FRET efficiencies greater than about 0.8 for ML and LS analysis, and at about 0.9 for Bayesian analysis (data where the bias is greater than 5% has not been plotted).

The sensitivity to different interacting fractions is shown in (Fig 4c and 4f) for synthetic data having a FRET efficiency of *E*^⋆^ = 0.75 (i.e. $\tau_{1}^{⋆}=2.0$ ns, $\tau_{2}^{⋆}=0.5$ ns) and an intensity of about 10^4^ total counts. Bayesian analysis offers a modest improvement in precision over ML and LS analysis in this instance.

Of course, some bi-exponential decays are more amenable to accurate analysis than others; it would be reasonable to expect it to be more difficult to resolve the two decay components if they have similar lifetimes (i.e. a low FRET efficiency) and impossible at the point where they are equal (FRET efficiency equal to zero) or if one of the components dominates the decay (i.e. a very high or very low interacting fraction). It is these competing effects that give rise to the minima in (Fig 4b and 4e), the position of the minimum depending on the level of interacting fraction. The reader may note that in this example values of *E* < 0.5 are not well estimated. This situation becomes better with different interacting fractions and more photon counts, but it should be remembered that this attempts to extract the actual FRET efficiency and not the effective FRET efficiency often reported.

Bayesian analysis offers superior estimation of biologically relevant quantities in many situations such as the FRET efficiency and interacting fraction in comparison to ML and LS analysis. Although the improvement in precision using Bayesian analysis is modest compared to ML and LS analysis, in some cases this improvement does mean that fewer total photon counts are required for analysis at a given precision or that a wider range of FRET efficiency or interacting fractions can be studied. It may be that a modest improvement in the precision of the estimated FRET efficiency could be the difference between observing a statistically significant difference in an experiment in which only a small change in the FRET efficiency is expected. It is also worth noting that Bayesian analysis in this form is very rarely worse than the other methods, and this is true at higher photon counts, so the only possible penalty from exclusively employing the Bayesian analysis is increased analysis time.

### Bayesian decay model selection

Typically, a combination of factors influence which decay model is chosen to analyse the experimental data. In choosing the decay model the expectation of what form the decay is *believed* to be is always likely to be significant, as is whether the intensity of the acquired experimental data is sufficient to support a statistically significant analysis using higher-order models. The application of the selected model is often justified by inspecting the parameter estimates, and the closeness of the fits to the acquired data at a representative sample of image pixels. Closeness can be assessed by examining the residual distance (difference) between the fit and the data, and even the auto-correlation of the point-wise residuals [37].

The Bayesian decay model selection analysis algorithm (Eq (24)) can be applied to determine probabilistically the decay model that most-likely underlies the time-resolved data from *any number* of different models. In this section the results of a Bayesian decay model selection algorithm, *chosen* to distinguish between mono- and bi-exponential decay data, are shown; the developed algorithm determines the most-probable decay model $H^{⋆}$ from an ensemble containing only the mono-exponential decay model $H_{1}$ and bi-exponential decay model $H_{2}$ (the mono- and bi-exponential decay models being formalised according to Eq (9) with *K* = 1 and *K* = 2 respectively).

**Fig 5:**
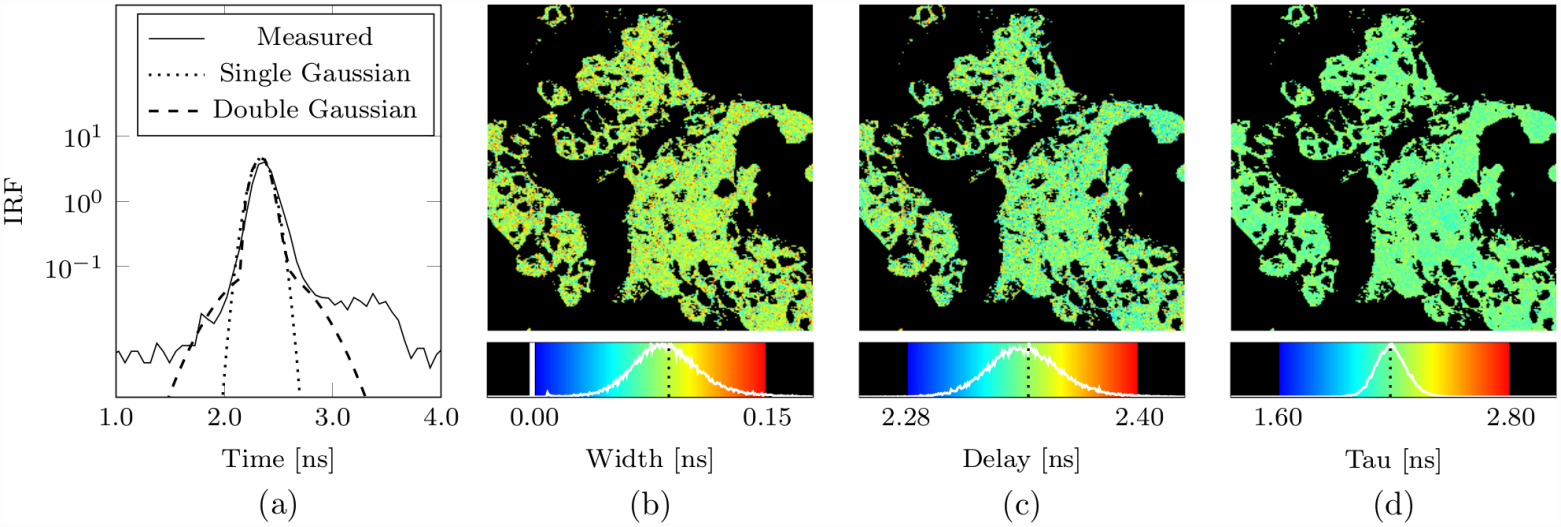
Bayesian decay model selection. In (a) an intensity image having pixels with intensity of about 750 photon counts, those on the left half of the image simulating a mono-exponential decay and those on the right half of the image simulating a bi-exponential decay; in (b) and (c) the Bayesian determined probability of the decay model being bi-exponential, $p(H_{2})$, and the optimal decay model, $H^{⋆}$, respectively, and in (d) the optimal model as determined by the *χ*^2^ model selection algorithm of [38] using the ML parameter estimates. Similar images are shown for human cancer cells expressing GFP (e-h), showing largely mono-exponential characteristics, and for human breast cancer tissue (i-l) which has many ‘contaminants’ from heterogeneous tissue types giving rise to bi-exponential, or higher order, responses. See main text for details.

The results of model selection are shown in Fig 5 alongside those from the *χ*^2^ model selection of [38] acting on the ML analysis, for the analysis of an image comprising both mono- and bi-exponential synthetic data, an image of the GFP-expressing cells expected to follow a mono-exponential decay (see Appendix A3 Human Epithelial Carcinoma Cell Preparation), and the human breast cancer tissue image (see Appendix A5 Human Breast Cancer Tissue Preparation).

The Bayesian model selection algorithm is able to successfully distinguish between mono-exponential and bi-exponential decay data at very low intensities of about 750 photon counts, as shown in (Fig 5b and 5c). The pixels in the left half of the image contain mono-exponential decay data, each generated to have a decay lifetime of $\tau_{1}^{⋆}=2.0\;\text{ns}$, and the pixels in the right half contain bi-exponential decay data, each generated to have one decay component with lifetime $\tau_{1}^{⋆}=2.0\;\text{ns}$ and another with lifetime $\tau_{2}^{⋆}=0.5\;\text{ns}$, their initial amplitudes being equal. The analysed transients were generated to simulate our TCSPC system (repetition rate: 40 MHz, measurement interval: 20.0 ns partitioned into 256 equal bins) and incorporated the effects of a Gaussian instrument response, no background (i.e. uniform background of zero counts per bin), and the effects of Poisson noise. Of the 2048 mono-exponential decays that were analysed 84 of them were incorrectly classified as being bi-exponential (i.e. 4.1% false positive) and of the 2048 bi-exponential decays analysed 53 were classified as being mono-exponential (i.e. 2.6% false negative). Overall, the occurrence of FRET was predicted with about 97% accuracy by Bayesian decay model selection. The *χ*^2^ based model selection algorithm predicted only 89% of the mono-exponential decays correctly, and the performance on bi-exponential data is visibly poorer.

The results of application of Bayesian and *χ*^2^-ML decay model selection to the GFP-expressing human carcinoma cell data (see Appendix A3 Human Epithelial Carcinoma Cell Preparation) are shown in (Fig 5f, 5g and 5h); both methods demonstrate that the image largely consists of mono-exponential decay data as should be expected. There is correlation between a region of high intensity pixels and a bi-exponential decay being determined as optimal by the *χ*^2^-ML decay model selection algorithm, as evident on inspection of (Fig 5h); it is suspected that the *χ*^2^-ML decay model selection may be biased towards selection of the bi-exponential decay model as a consequence of poor decay estimates compensating for an underestimation of the background level by ML analysis at these pixels.

In (Fig 5j, 5k and 5l) the data from a human breast cancer tissue (see Appendix A5 Human Breast Cancer Tissue Preparation) image is predicted to follow a bi-exponential decay as would be consistent with expectation. Tissue of this type has numerous ‘contaminants’ from heterogeneous tissue types, endogenous fluorophores and preparation chemicals that will give rise to bi-exponential, or higher order, responses. This binary ‘mono’ or ‘bi’ selection algorithm will be more likely to assign higher order exponential responses into the bi-exponential category as a the better explanation of the data.

It is important to appreciate that in interpreting the results of Bayesian model selection, inferences can only be made concerning those models that are present in the ensemble. The algorithms as applied in this work operate over an ensemble consisting only of mono- and bi-exponential decays; and it is therefore not possible to make any inference as to whether the data may actually be more likely to be due to, for example, a tri-exponential or even some non-exponential decay process. However, the Bayesian algorithm could be extended to offer the relative likelihoods of mono-, bi-, and tri-exponential decays. Similarly, a background noise only model could be incorporated to permit determination of the presence or absence of a decay signal in some data, along the lines of the method realised in [39].

### Bayesian IRF determination

The Bayesian IRF determination algorithm has been developed according to Eq (28) such that the parameters describing the optimal IRF approximation *and* the decay parameters are estimated simultaneously from the TCSPC data: Simultaneous IRF and Decay (SID) analysis. As both sets of parameters are estimated simultaneously the algorithm may be applied for either purpose under different experimental conditions.

Typically, the Bayesian IRF determination algorithm will be run over a data set having very high photon counts in order to estimate the optimal IRF approximation to be used in subsequent decay analysis; in this instance, the decay parameter estimates would be of little interest. An alternative application of the Bayesian IRF determination algorithm, however, could be to provide *decay* parameter estimates from a system for which the IRF changes greatly between pixels; in this instance, it would be the IRF approximation parameters that may be of little interest.

**Fig 6:**
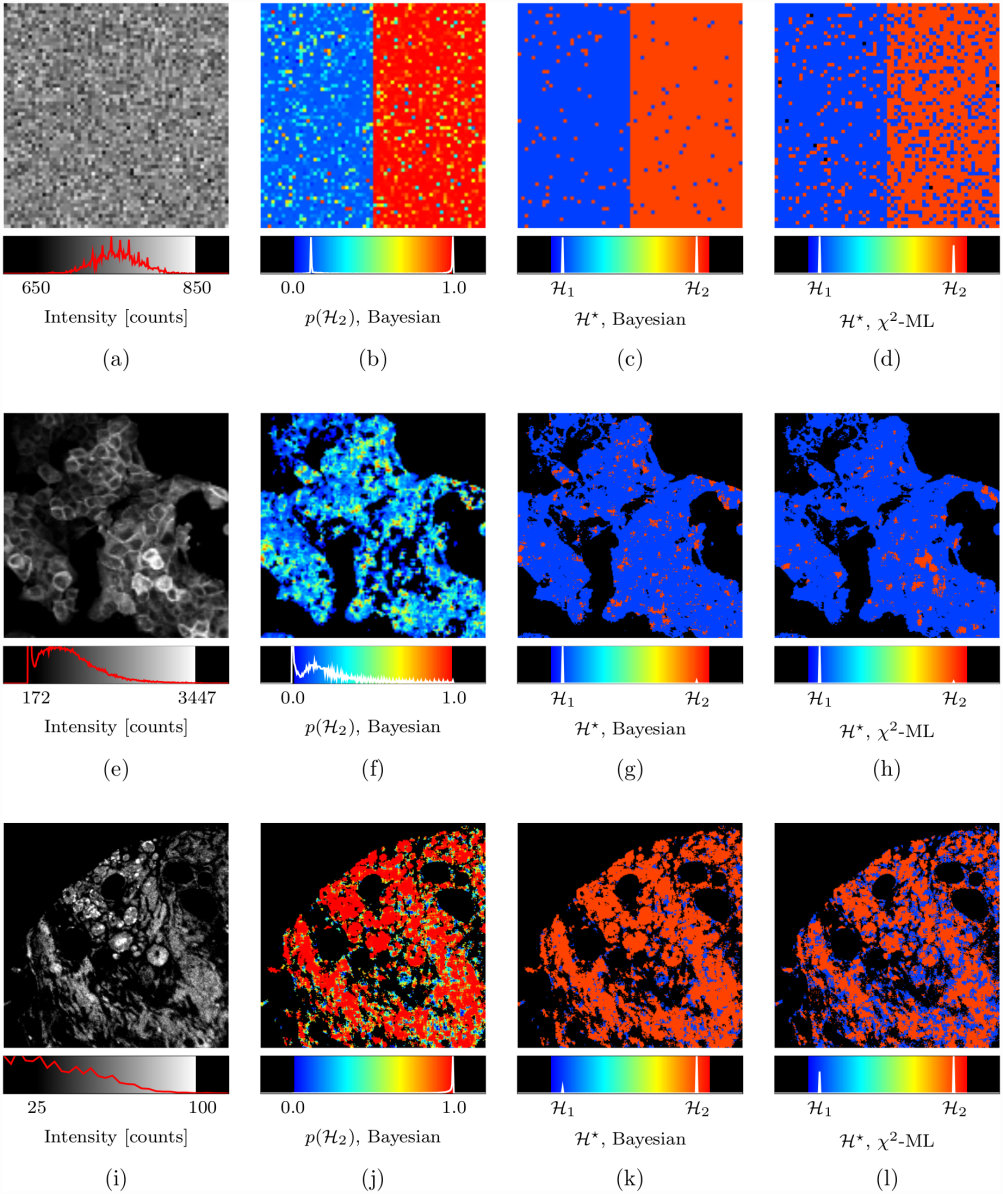
Bayesian simultaneous decay and IRF analysis. In (a) the measured and optimal single- and double-Gaussian IRF approximations as determined on application of the Bayesian SID algorithm to a single data set having over 10^7^ counts obtained by binning the data from all image pixels from a single image of the human carcinoma cell data (see Appendix A3 Human Epithelial Carcinoma Cell Preparation). In (b) and (c), the width and delay parameter estimates obtained on independent analysis of each pixel of the same human carcinoma cell data image using the Bayesian SID algorithm, assuming a mono-exponential decay and a single-Gaussian approximation, for pixels having intensities between about 350 counts and 3500 counts; in (d) the corresponding lifetime estimates. In (b), (c), and (d), the corresponding optimal single-Gaussian IRF approximation estimates are indicated in the histograms by a dotted black line.

The effectiveness of the Bayesian IRF determination algorithm to provide accurate decay and IRF approximation parameter estimates simultaneously is demonstrated in Fig 6 for human carcinoma cell data (see Appendix A3 Human Epithelial Carcinoma Cell Preparation).

The optimal single- and double-Gaussian IRF approximations for these data are shown along with the measured IRF in (Fig 6a); these estimates were obtained on analysis of a single data set having over 10^7^ counts, obtained by binning the data from all image pixels from an image having intensities between about 350 and 3500 counts. The optimal single-Gaussian IRF approximation is defined by the estimates $I_{1}=\left\{\gamma_{1}=1.000,\delta_{1}=0.037\;\text{ns},u_{1}=2.341\;\text{ns},\sigma_{1}=0.087\;\text{ns}\right\}$; the optimal double-Gaussian IRF approximation is defined by $I_{2}=\left\{\gamma_{1}=0.931,\delta_{1}=2.167\;\text{ns},u_{1}=2.336\;\text{ns},\sigma_{1}=0.079\;\text{ns},\gamma_{2}=0.069,\delta_{2}=0.855\;\text{ns},u_{2}=2.395\;\text{ns},\sigma_{2}=0.302\;\text{ns}\right\}$. It is evident that the double-Gaussian approximation follows the measured IRF more closely at either side of the main peak than does the single-Gaussian approximation.

The single-Gaussian IRF approximation width and delay estimates obtained on independent application of the Bayesian SID algorithm, under the assumption of a mono-exponential decay, to each pixel are shown in (Fig 6b, 6c and 6d), for pixels with intensities between about 350 and 3500 counts, with most pixels having an intensity of less than 1000 counts.

The distribution of the delay estimates has an average of 2.342 ns and a standard deviation of 0.019 ns, and the width parameter estimates are distributed around an average value of 0.086 ns with a standard deviation of 0.022 ns; both are in close agreement with the optimal values determined using the high count data set. The mono-exponential lifetime estimates obtained on application of the Bayesian SID are inferior to those obtained on applying Bayesian decay analysis with the optimal IRF approximation, as might be expected given that the IRF parameters are additionally estimated from the same data. However, for such decay data and for such an instrument for which a single Gaussian serves as a reasonable approximation of the IRF, it is noteworthy that the lifetime estimates are only slightly degraded in comparison to those obtained from Bayesian decay analysis alone using the optimal approximated IRF, so there remains a significant improvement over the estimates offered by ML (which uses the experimentally measured IRF). The Bayesian SID lifetime distribution is centered about an average of 2.18 ns with standard deviation 0.17 ns. The estimates derived from Bayesian decay analysis using the optimal IRF approximation have an average of 2.16 ns and a standard deviation of 0.15 ns. The ML estimates (lifetime image not shown) were centered about an average lifetime of 2.17 ns with standard deviation 0.25 ns.

## Discussion

In this paper we have presented the novel analysis of exponential fluorescence decay data using Baysian inference that is based on the concept that each single photon carries some evidence about the photo-physical system from which it was emitted. In particular, algorithms that relate to the analysis of time-domain FLIM data from microscopes have been presented which may be used in the microscopical studies of cellular protein interactions employing FRET. We have extended our previous work on mono-exponential analysis [18], that indicated the possible doubling of the acquisition speed is possible by using the Bayesian approach, to include the following important advances:

- complex decay modelling, demonstrated here by bi-exponential analysis
- simultaneous estimation of additional system parameters such as the IRF
- decay model selection
- rigorous treatment of repetitive pulse excitation
- complex IRF modelling with multiple Gaussians

We have shown that this novel analysis performs better than the two most popular methods of ‘direct fitting’, LS and ML, in terms of accuracy and bias, and offers a distinct advantage when photon counts are low and the TCSPC-accumulated histogram is not a good representation of the underlying fluorescence decays. The greatest improvement over previous techniques was seen when performing mono-exponential analysis, where a factor of two improvement in accuracy was observed. Since time-domain FLIM using TCSPC is a relatively slow method of acquisition, this factor of two improvement can be exploited to image significantly faster for the same accuracy as previously. This may allow time-lapse experiments where imaging speed is important. The improvement due to the new algorithms when performing bi-exponential analysis was less impressive, but still offers a significantly better accuracy in determined parameters, extending the range of FRET parameters that can be accommodated and therefore the biological protein interactions that can be studied.

The analysis has also been extended to include model selection to infer robustly whether underlying photo-physical system(s) have a single fluorescence lifetime or would be better represented by a bi-exponential model. This type of analysis is not common in the FLIM field and as well as indicating which type of model to use, it can also be used to indicate to the user whether there data is of sufficient quality (e.g. contains sufficient photon counts) to provide robust parameter estimates of a high-order model; a common pitfall for the novice FLIM user.

We also presented the first published simultaneous estimate of the IRF and decay parameters based only on the assumption of the IRF being approximated by a mixture of Gaussian distributions. This represents a step forward in determining dynamically the response of the instrument as well as the sample. Although it is not impossible to obtain a reasonable estimate of the IRF for many FLIM systems experimentally, obtaining parameter estimates algorithmically has advantages. One is that it enables us to use analytical expressions that aid implementation and execution speed. A second and more generally applicable advantage is relevant to the use of other types of fluorescence lifetime equipment (or other ‘time-of-flight’ systems [40, 41]) where it may be impossible to determine the IRF, or where the IRF may change from pixel to pixel of the image (e.g. when imaging natural scenes the distance to the sample introduces a variable delay onto the signal).

All the algorithms are available for use in the TRI2 image processing program. This is currently available as compiled ‘freeware’ for Windows from the Oxford Group’s web site (http://users.ox.ac.uk/~atdgroup/software/).

## Appendix

### A1 Mathematical Details

**Development of the time domain FLIM model** Intermediate steps required for the development of our time-domain FLIM system model that were not presented in the main paper are detailed below along with a brief commentary as to the mathematical properties employed at each stage.

The development of the signal photon-likelihood, from the integral form defined by Eq (4) to the more generally useful form of Eq (5) where the influence of recording arrival times with respect to a periodic window is captured by a summation, is now presented. The integral in Eq (4) can be rewritten to give

$$\int_{0}^{\infty }du\;\Gamma \left(u\right)\;\delta \left(\Delta t-t-u+T_{m}.\;\left\lfloor \frac{t+u}{T_{m}}\right\rfloor \right)$$ (29)

$$=\sum_{\ell \;\geq 0}\int_{0}^{\infty }du\;\Gamma \left(u\right)\;\delta \left(\Delta t-t-u+\ell T_{m}\right)\;\delta_{\ell ,\;\left\lfloor \left(t+u\right)/T_{m}\right\rfloor }$$ (30)

$$=\sum_{\ell \;\geq 0}\Gamma \left(\Delta t-t+\ell T_{m}\right)\int_{0}^{\infty }du\;\;\delta \left(\Delta t-t-u+\ell T_{m}\right)\;\delta_{\ell ,\;\left\lfloor \left(t+u\right)/T_{m}\right\rfloor }$$ (31)

$$=\sum_{\ell \;\geq 0}\Gamma \left(\Delta t-t+\ell T_{m}\right)\;\theta \left[\Delta t-t+\ell T_{m}\right]\;\delta_{\ell ,\;\left\lfloor \left(\Delta t+\ell T_{m}\right)/T_{m}\right\rfloor }$$ (32)

$$=\sum_{\ell \;\geq 0}\Gamma \left(\Delta t-t+\ell T_{m}\right)\delta_{\ell ,\;\left\lfloor \left(\Delta t+\ell T_{m}\right)/T_{m}\right\rfloor }\;\theta \left[\ell +\left(\Delta t-t\right)/T_{m}\right]$$ (33)

where *δ*(*x*) represents the Dirac delta function which exists only when *x* is equal to zero (i.e. ∫*dx* *δ*(*x*)*f*(*x*) = *f*(0) for any function *f*(*x*)), *δ*_*i*,*j*_ is the Kronecker delta function which exists only when *i* = *j*, the step function is denoted by *θ*(*x*) with *θ*(*x* > 0) = 1 and *θ*(*x* ≤ 0) = 0, and the floor function is denoted by ⌊*x*⌋, such that *n* = ⌊*x*⌋ is the largest integer *n* ≤ *x*. By definition, any observed photon arrival time Δ*t* ∈ [0, *T*] must fall within the measurement interval and therefore cannot exceed the modulation period *T*_*m*_ (i.e. Δ*t* ≤ *T* ≤ *T*_*m*_), consequently *δ*_*ℓ*,⌊*ℓ*+Δ*t*/*T*_*m*_⌋_ = *δ*_*ℓ*,*ℓ*_ = 1 and the integral simplifies further to give

$$\begin{array}{cc} & \int_{0}^{\infty }du\;\Gamma \left(u\right)\delta \left(\Delta t-t-u+T_{m}.\text{int}\left(\frac{t+u}{T_{m}}\right)\right)\\ & \;=\sum_{\ell \geq 0}\Gamma \left(\Delta t-t+\ell T_{m}\right)\theta \left[\Delta t-t+\ell T_{m}\right] \end{array}$$ (34)

Since the instrument response approximation Γ(*u*) is non-zero only for non-negative delay times *u* (i.e. Γ(*u*) = 0 if *u* < 0), the above expression simplifies yet further to be of the form in Eq (5), that is

$$\begin{array}{cc} & \int_{0}^{\infty }du\;\Gamma \left(u\right)\delta \left(\Delta t-t-u+T_{m}.\text{int}\left(\frac{t+u}{T_{m}}\right)\right)=\sum_{\ell \geq 0}\Gamma \left(\Delta t-t+\ell T_{m}\right). \end{array}$$ (35)

Incorporating the discrete-time nature of these time-domain FLIM data into our model to give the photon and signal photon likelihood in discrete time, *p*(*b*) and *S*(*b*), as given by Eqs (7) and (8) respectively, is trivial, requiring that the photon and signal photon likelihoods in continuous time, *p*(Δ*t*) and *S*(Δ*t*), as given by Eqs (3) and (5) respectively, be integrated over the interval defining the bin *b* = [*b*^L^, *b*^H^]. The introduction of a multi-exponential fluorescence decay signal of the form defined by Eq (9), and subject to the constraints of Eq (10), leads to the photon bin-likelihood, *p*(*b*), as given by Eq (11), it now being convenient to define the fluorescence bin-likelihood, $F(\tau ,b^{L},b^{H},I)$, which describes the likelihood of a fluorescence decay photon arrival time being counted in the interval *b* = [*b*^L^, *b*^H^] due to a mono-exponential decay with lifetime *τ*, as given by Eq (12).

The introduction of an approximation to the IRF, Γ(u,I), comprising a weighted sum of truncated Gaussian distributions and of the form described by Eq (13), leads to the fluorescence decay bin-likelihood as given by Eq (19) where the necessary integrals are performed analytically, as described below. The IRF approximation can be written as $\Gamma \left(u,I\right)=\sum_{i=1}^{I}{\tilde{\Gamma }}_{i}\left(u,I\right)$, where the contribution of the *i*th IRF component, denoted by ${\tilde{\Gamma }}_{i}\left(u,I\right)$, is given by

$$\begin{array}{c} {\tilde{\Gamma }}_{i}\left(u,I\right)=\frac{\sqrt{2}}{\sqrt{\pi }}\frac{\tilde{\gamma_{i}}}{\sigma_{i}}\;e^{-\frac{1}{2}{\left(u-u_{i}\right)}^{2}/\sigma_{i}^{2}}\;\theta \left[u-\delta_{i}\right] \end{array}$$ (36)

where the quantity $l\tilde{\gamma_{i}}=\gamma_{i}{\left(1+\text{erf}((u_{i}-\delta_{i})/\sigma_{i}\sqrt{2}))\right)}^{-1}$ is introduced for compactness. The fluorescence decay bin-likelihood, as given in Eq (12), can then be decomposed into the sum of contributions of each of the instrument response components, such that $F\left(\tau ,b^{L},b^{H},I\right)=\sum_{i}{\tilde{F}}_{i}\left(\tau ,b^{L},b^{H},I\right)$, where ${\tilde{F}}_{i}\left(\tau ,b^{L},b^{H},I\right)$ represents the contribution of the *i*th instrument response component to the overall fluorescence decay bin-likelihood, and is given by

$$\begin{array}{c} {\tilde{F}}_{i}\left(\tau ,b^{L},b^{H},I\right)=\frac{\tau^{-1}}{\Lambda (T,T_{m})}\int_{b^{L}}^{b^{H}}d\Delta t\sum_{\ell \geq 0}\int_{0}^{\infty }dt\;e^{-t/\tau }\;{\tilde{\Gamma }}_{i}\left(\ell T_{m}+\Delta t-t\right) \end{array}$$ (37)

Determining now the convolution of a component of the multi-exponential decay signal, having a decay lifetime *τ*, and the *i*th component of the instrument response approximation as follows:

$$\begin{array}{cc} & \int_{0}^{\infty }dt\;e^{-t/\tau }\;{\tilde{\Gamma }}_{i}\left(\ell T_{m}+\Delta t-t\right)\\ & \;=\frac{\sqrt{2}}{\sqrt{\pi }}\;\frac{\tilde{\gamma_{i}}}{\sigma_{i}}\;\int_{0}^{\infty }dt\;e^{-\frac{1}{2}{\left(\ell T_{m}+\Delta t-t-u_{i}\right)}^{2}/\sigma_{i}^{2}-t/\tau }\;\theta \left[\ell T_{m}+\Delta t-t-\delta_{i}\right]\\ & \;=\frac{\sqrt{2}}{\sqrt{\pi }}\;\frac{\tilde{\gamma_{i}}}{\sigma_{i}}\;\theta \left[\ell T_{m}+\Delta t-\delta_{i}\right]\;\int_{0}^{\ell T_{m}+\Delta t-\delta_{i}}dt\;e^{-\frac{1}{2}{\left(\ell T_{m}+\Delta t-t-u_{i}\right)}^{2}/\sigma_{i}^{2}-t/\tau }\\ & \;=\tilde{\gamma_{i}}\;e^{u_{i}/\tau +\sigma_{i}^{2}/2\tau^{2}}\;\theta \left[\ell T_{m}+\Delta t-\delta_{i}\right]\;{\tilde{\chi }}_{i}\left(\ell ,\Delta t,\tau ,T_{m},\sigma_{i},\delta_{i},u_{i}\right) \end{array}$$ (38)

where the quantity ${\tilde{\chi }}_{i}\left(\ell ,\Delta t,\tau ,T_{m},\sigma_{i},\delta_{i},u_{i}\right)$ is given by

$$\begin{array}{cc} & {\tilde{\chi }}_{i}\left(\ell ,\Delta t,\tau ,T_{m},\sigma_{i},\delta_{i},u_{i}\right)\\ & =e^{-(\ell T_{m}+\Delta t)/\tau }\left[\text{erf}\left(\frac{\left(u_{i}-\delta_{i}\right)\tau +\sigma_{i}^{2}}{\sigma_{i}\tau \sqrt{2}}\right)-\text{erf}\left(\frac{\left(u_{i}-\ell T_{m}-\Delta t\right)\tau +\sigma_{i}^{2}}{\sigma_{i}\tau \sqrt{2}}\right)\right] \end{array}$$

Notice that the term *θ*[*ℓT*_*m*_ + Δ*t* − *δ*_*i*_] ensures that the integral is positive. The developed expression describes (without normalisation) the likelihood of a fluorescence decay photon at time Δ*t* in the measurement interval. The fluorescence decay bin-likelihood due to the *i*th instrument response component can now be written as

$$\begin{array}{cc} & {\tilde{F}}_{i}\left(\tau ,b^{L},b^{H},I\right)\\ & =\frac{\tau^{-1}}{\Lambda (T,T_{m})}\tilde{\gamma_{i}}\;e^{u_{i}/\tau +\sigma_{i}^{2}/2\tau^{2}}\;\sum_{\ell \geq 0}\int_{b^{L}}^{b^{H}}d\Delta t\;{\tilde{\chi }}_{i}\left(\ell ,\Delta t,\tau ,T_{m},\sigma_{i},\delta_{i},u_{i}\right)\;\theta \left[\ell T_{m}+\Delta t-\delta_{i}\right] \end{array}$$ (39)

Observe that the term *θ*[*ℓT*_*m*_ + Δ*t* − *δ*_*i*_] ensures that the fluorescence photon likelihood is zero until the decay time *ℓT*_*m*_ + Δ*t* exceeds the cutoff parameter *δ*_*i*_. In determining the remaining integral, which accounts for the discrete time nature of TCSPC data, it is convenient to incorporate the bin boundaries directly into the developed expression, to give

$$\begin{array}{cc} & \int_{b^{L}}^{b^{H}}d\Delta t\;{\tilde{\chi }}_{i}\left(\ell ,\Delta t,\tau ,T_{m},\sigma_{i},\delta_{i},u_{i}\right)\;\theta \left[\ell T_{m}+\Delta t-\delta_{i}\right]\\ & \;=\int_{0}^{\infty }d\Delta t\;{\tilde{\chi }}_{i}\left(\ell ,\Delta t,\tau ,T_{m},\sigma_{i},\delta_{i},u_{i}\right)\;\theta \left[\Delta t-\left(\delta_{i}-\ell T_{m}\right)\right]\theta \left[\Delta t-b^{L}\right]\theta \left[b^{H}-\Delta t\right]\\ & \;=\left\{\begin{array}{cc} 0, & \text{if}\;b^{L}\leq b^{H}\leq \delta_{i}-\ell T_{m}\\ \int_{\delta_{i}}^{b^{H}}d\Delta t\;{\tilde{\chi }}_{i}\left(\ell ,\Delta t,\tau ,T_{m},\sigma_{i},\delta_{i},u_{i}\right), & \text{if}\;b^{L}\leq \delta_{i}-\ell T_{m}\leq b^{H}\\ \int_{b^{L}}^{b^{H}}d\Delta t\;{\tilde{\chi }}_{i}\left(\ell ,\Delta t,\tau ,T_{m},\sigma_{i},\delta_{i},u_{i}\right), & \text{if}\;\delta_{i}-\ell T_{m}\leq b^{L}\leq b^{H} \end{array}\right. \end{array}$$ (40)

It is evident that if the time bin lies entirely before the cutoff there is no likelihood of a fluorescence decay photon being counted into it, the likelihood of photon being counted into a time bin which straddles the cutoff is determined by integrating between the cutoff and the upper bin boundary, and if the time bin is entirely beyond the cutoff then the likelihood of a photon arrival time in the bin is determined by integrating between the bin boundary values. These conditions are encapsulated by the following expression,

$$\begin{array}{cc} & \int_{b^{L}}^{b^{H}}d\Delta t\;\tilde{\chi }\left(\ell ,\Delta t,\tau ,T_{m},\sigma_{i},\delta_{i},u_{i}\right)\theta \left[\ell T_{m}+\Delta t-\delta_{i}\right]\\ & \;=\theta \left[\ell T_{m}+b^{L}-\delta_{i}\right]\int_{b^{L}}^{\delta_{i}}d\Delta t\;\tilde{\chi }\left(\ell ,\Delta t,\tau ,T_{m},\sigma_{i},\delta_{i},u_{i}\right)\\ & \;+\theta \left[\ell T_{m}+b^{H}-\delta_{i}\right]\int_{\delta_{i}}^{b^{H}}d\Delta t\;\tilde{\chi }\left(\ell ,\Delta t,\tau ,T_{m},\sigma_{i},\delta_{i},u_{i}\right) \end{array}$$ (41)

The remaining integral is determined analytically, by parts, giving

$$\begin{array}{cc} & \int d\Delta t\;e^{-(\ell T_{m}+\Delta t)/\tau }\left[\text{erf}\left(\frac{\left(u_{i}-\delta_{i}\right)\tau +\sigma_{i}^{2}}{\sigma_{i}\tau \sqrt{2}}\right)-\text{erf}\left(\frac{\left(u_{i}-\ell T_{m}-\Delta t\right)\tau +\sigma_{i}^{2}}{\sigma_{i}\tau \sqrt{2}}\right)\right]\\ & =\tau \;e^{-(\ell T_{m}+\Delta t)/\tau }\left[\text{erf}\left(\frac{\left(u_{i}-\ell T_{m}-\Delta t\right)\tau +\sigma_{i}^{2}}{\sigma_{i}\tau \sqrt{2}}\right)-\text{erf}\left(\frac{\left(u_{i}-\delta_{i}\right)\tau +\sigma_{i}^{2}}{\sigma_{i}\tau \sqrt{2}}\right)\right]\\ & \;-\tau \;e^{-\sigma_{i}^{2}/2\tau^{2}-u_{i}/\tau }\;\text{erf}\left(\frac{u_{i}-\ell T_{m}-\Delta t}{\sigma_{i}\sqrt{2}}\right) \end{array}$$ (42)

Defining the quantity,

$$\begin{array}{cc} & \chi (\ell ,t,\tau ,T_{m},\sigma_{i},\delta_{i},u_{i})\\ & =e^{-\left(\ell T_{m}+t-u_{i}\right)/\tau +\sigma_{i}^{2}/2\tau^{2}}\left[\text{erf}\left(\frac{\left(u_{i}-\ell T_{m}-t\right)\tau +\sigma_{i}^{2}}{\sigma_{i}\tau \sqrt{2}}\right)-\text{erf}\left(\frac{\left(u_{i}-\delta_{i}\right)\tau +\sigma_{i}^{2}}{\sigma_{i}\tau \sqrt{2}}\right)\right]\\ & \;-\text{erf}\left(\frac{u_{i}-\ell T_{m}-t}{\sigma_{i}\sqrt{2}}\right) \end{array}$$ (43)

the integral can be written (using Eq (41)), whilst also absorbing the factor $\tau^{-1}\;e^{u_{i}/\tau +\sigma_{i}^{2}/2\tau^{2}}$ from Eq (39), to give

$$\begin{array}{cc} & \tau^{-1}\;e^{u_{i}/\tau +\sigma_{i}^{2}/2\tau^{2}}\int_{b^{L}}^{b^{H}}d\Delta t\;{\tilde{\chi }}_{i}\left(\ell ,t,\tau ,T_{m},\sigma_{i},\delta_{i},u_{i}\right)\theta \left[\ell T_{m}+\Delta t-\delta_{i}\right]\\ & \;=\theta \left[\ell T_{m}+b^{L}-\delta_{i}\right]\left\{\chi \left(\ell ,\delta_{i},\tau ,T_{m},\sigma_{i},\delta_{i},u_{i}\right)-\chi \left(\ell ,b^{L},\tau ,T_{m},\sigma_{i},\delta_{i},u_{i}\right)\right\}\\ & \;+\theta \left[\ell T_{m}+b^{H}-\delta_{i}\right]\left\{\chi \left(\ell ,b^{H},\tau ,T_{m},\sigma_{i},\delta_{i},u_{i}\right)-\chi \left(\ell ,\delta_{i},\tau ,T_{m},\sigma_{i},\delta_{i},u_{i}\right)\right\} \end{array}$$ (44)

Consequently, the fluorescence decay bin-likelihood due to the *i*th instrument response component can be written as follows:

$$\begin{array}{cc} & {\tilde{F}}_{i}\left(\tau ,b^{L},b^{H},T,T_{m},I\right)=\frac{\tilde{\gamma_{i}}}{\Lambda (T,T_{m})}\;\sum_{\ell \geq 0}\;\Psi_{i}\left(\tau ,b^{L},b^{H},T,T_{m},\sigma_{i},\delta_{i},u_{i}\right) \end{array}$$ (45)

where the quantity Ψ*_i_* (*τ*, *b^L^*, *b^H^*, *T*, *T_m_*, *σ_i_*, *δ_i_*, *u_i_*) is given by

$$\begin{array}{cc} & \Psi_{i}\left(\tau ,b^{L},b^{H},T,T_{m},\sigma_{i},\delta_{i},u_{i}\right)=\\ & \;\theta \left[\ell T_{m}+b^{L}-\delta_{i}\right]\left\{\chi \left(\ell ,\delta_{i},\tau ,T_{m},\sigma_{i},\delta_{i},u_{i}\right)-\chi \left(\ell ,b^{L},\tau ,T_{m},\sigma_{i},\delta_{i},u_{i}\right)\right\}\\ & \;+\theta \left[\ell T_{m}+b^{H}-\delta_{i}\right]\left\{\chi \left(\ell ,b^{H},\tau ,T_{m},\sigma_{i},\delta_{i},u_{i}\right)-\chi \left(\ell ,\delta_{i},\tau ,T_{m},\sigma_{i},\delta_{i},u_{i}\right)\right\} \end{array}$$ (46)

Incorporating this into the model yields the following expression for the bin-likelihood (which includes the contribution of both background and a fluorescence decay signal):

$$p(b)=\left|b\right|\frac{w_{0}}{T}+\sum_{k=1}^{K}w_{k}F\left(\tau_{k},b^{L},b^{H},T,T_{m},I\right)$$ (47)

where $I=\{\gamma_{i},u_{i},\sigma_{i},\delta_{i}|i=1,\ldots ,I\}$ summarizes the parameters required for the approximation of the instrument response.

### A2 Synthetic Data

Synthetic data were generated to simulate a variety of mono- and bi-exponential decay conditions; all generated data reflected a modelled TCSPC system having a repetition rate of 40 MHz, a measurement interval of 20.0 ns partitioned into 256 bins of equal width, the effects of a Gaussian IRF having a FWHM width of 0.15 ns, and incorporated Poisson noise at each bin.

### A3 Human Epithelial Carcinoma Cell Preparation

Human epithelial *carcinoma* cells (A431) (ATTC, UK) stably expressing cdc42-GFP [42] were grown in Dulbecco’s modified Eagle’s medium containing 10% fetal calf serum. Cells were seeded on a glass coverslip (50000 cells/ml) and 48 hours later were fixed with 4% paraformaldehyde, treated with 1mg/ml NaBH4, then mounted with Mowiol (VWR, UK) containing antifade agent and kept at -20 degC.

### A4 Cell Pellet Preparation

Cell pellet preparations offer the opportunity to have the controllability of a cell experiment but in a medium that is similar to a tissue section. MCF7 cells (ATTC, UK) were cultured in DMEM supplemented with 10% FCS. Cells were transfected with HER2-GFP and HER3-GFP plasmids at ratio 3:1 using FuGene6 (Promega) according to the manufacturer’s protocol, and cultured for 24 hours. Cells were fixed with 10% formalin for 5 hours and then processed overnight on Leica ASP300S and embedded into paraffin on Leica EG11508C. For staining, cell pellets or tissue were cut (3 *μ*m) and underwent the same antigen retrieval procedure (BenchMark Ventana) and antibody-based staining with Alexa546 and Cy5.

### A5 Human Breast Cancer Tissue Preparation

FLIM data was obtained from an ongoing study of protein interactions in human tissue by our collaborators. Tissue microarrays were created from 218 primary breast cancers from patients included in the METABRIC (Molecular Taxonomy of Breast Cancer International Consortium) study [43], and whose material was stored by King’s Health Partners Cancer Biobank. These underwent standard de-waxing and antibody retrieval protocols, and were stained for HER3 and HER2 proteins. Anti-HER3 (B9A11) was purchased from Monogram Biosciences Inc., anti-HER2 (e2-4001+3B5) was purchased from ThermoScientific Ltd. and directly labeled according to the manufacturer’s protocol with Alexa546 (X546) and Cy5, respectively. Human tissue samples were provided by King’s Health Partners Cancer Biobank, a Human Tissue Authority licenced facility (reference 12121) with NHS Research Ethics Committee approval to provide samples for research (12/EE/0493).

### A6 FLIM Data Acquisition

Time-domain FLIM was performed with an in-house Open Microscope system [44], excitation being provided by a supercontinuum 40MHz source (SC450-4, Fianium, UK), pulse width ∼4 ps, and photon counting performed using a SPC830 TCSPC board (Becker & Hickl, Berlin, Germany). De-scanned detection was afforded by the use of a fast single-photon response, photomultiplier tube (PMH-100-0, Becker & Hickl, Berlin, Germany) placed behind a pinhole, parfocal with the image plane. The following filters were used in data acquisition from cell samples: 470 nm excitation filter (Semrock FF01-470/22-25, Laser 2000, UK) and an IR cut filter, 495 nm dichromatic reflector (Semrock FF 495-Di02-25x36, Laser 2000, UK), and 510 nm emission filter (Semrock FF01-510/20-25, Laser 2000, UK). The following filters were used in data acquisition from tissue samples: 540 nm excitation filter (Semrock FF01-540/15-25, Laser 2000, UK) and an IR cut filter, 30/70 neutral beam splitter (48NT-392 30R/70T, Edmund Optics, UK), and 593 nm emission filter (Semrock FF01-593/40-25, Laser 2000, UK)

To avoid pulse pile-up, peak photon counting rates were adjusted to be well below the maximum counting rate offered by the TCSPC electronics [34], with average photon counting rates of the order 10^4^ − 10^5^ photons/second. The photon arrival times, with respect to the 40 MHz repetitive laser pulses, were binned into 256 time windows over a total measurement period of 15 ns. Images were captured with a 0.75 NA objective lens (S Fluor 20x/0.75 air, Nikon, UK) at 256 × 256 pixels corresponding to 334 × 334 *μ*m at the sample. Data acquisition was performed at room temperature.

IRF measurement was performed by replacing the sample with an Aluminium-coated reflective slide and removing the emission filter such that reflected excitation light reaches the detector. In order to replicate experimental conditions in terms of laser power and photon detection rate, neutral density filters (ND10A and ND40A, Thor Labs, UK) were placed in the excitation light path.
