# Correction: Robust Bayesian Fluorescence Lifetime Estimation, Decay Model Selection and Instrument Response Determination for Low-Intensity FLIM Imaging

**DOI:** 10.1371/journal.pone.0162224

**Published:** 2016-08-25

**Authors:** Mark I. Rowley, Anthonius C. C. Coolen, Borivoj Vojnovic, Paul R. Barber

The images for Figs 5 and 6 are incorrectly switched. The image that appears as Fig 5 should be Fig 6, and the image that appears for Fig 6 should be Fig 5. The figure captions appear in the correct order.

**Fig 5:**
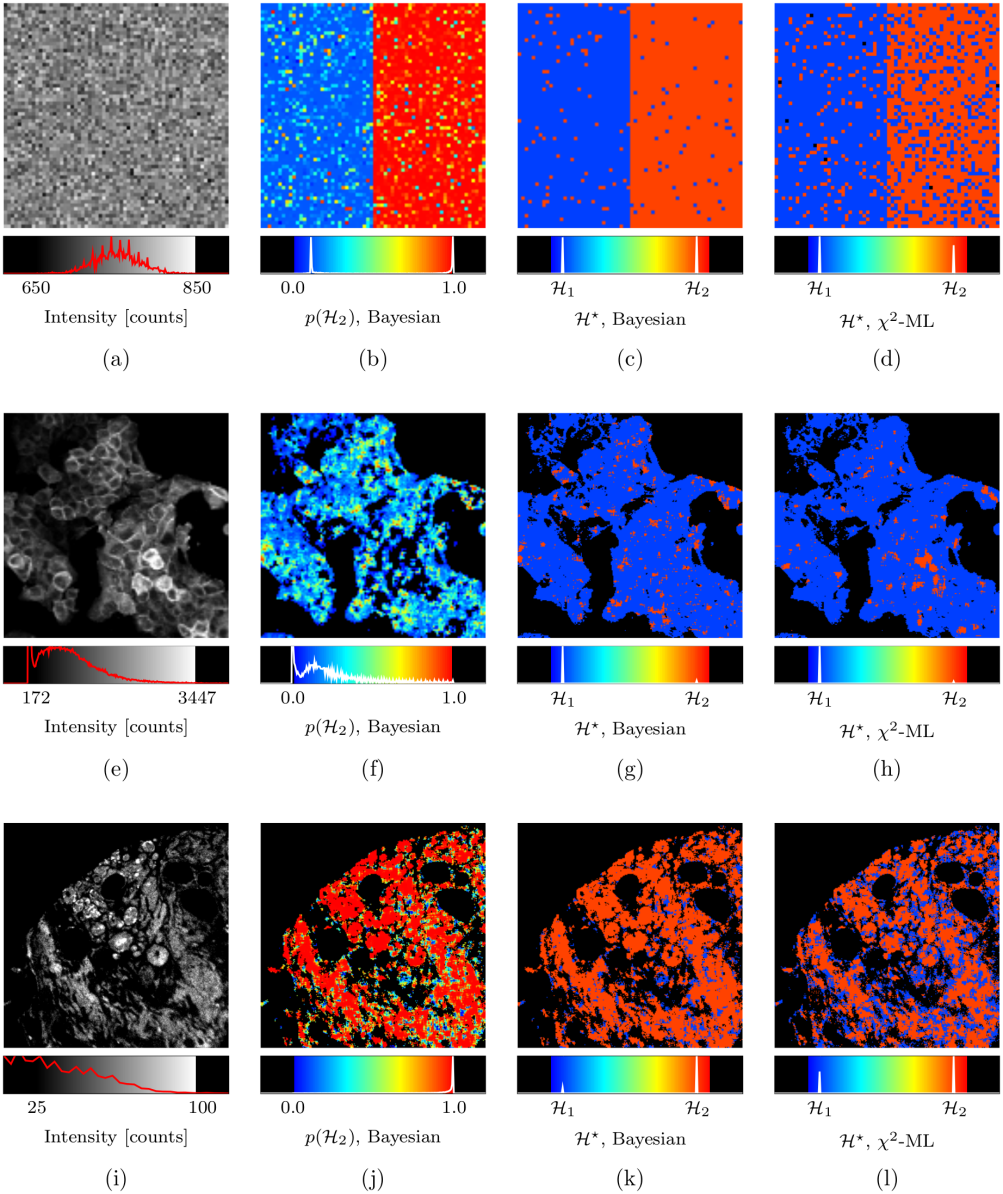
Bayesian decay model selection. In (a) an intensity image having pixels with intensity of about 750 photon counts, those on the left half of the image simulating a mono-exponential decay and those on the right half of the image simulating a bi-exponential decay; in (b) and (c) the Bayesian determined probability of the decay model being bi-exponential, p(ℋ_2_), and the optimal decay model, ℋ*, respectively, and in (d) the optimal model as determined by the *χ*^2^ model selection algorithm of [38] using the ML parameter estimates. Similar images are shown for human cancer cells expressing GFP (e-h), showing largely mono-exponential characteristics, and for human breast cancer tissue (i-l) which has many ‘contaminants’ from heterogeneous tissue types giving rise to bi-exponential, or higher order, responses. See main text for details.

**Fig 6:**
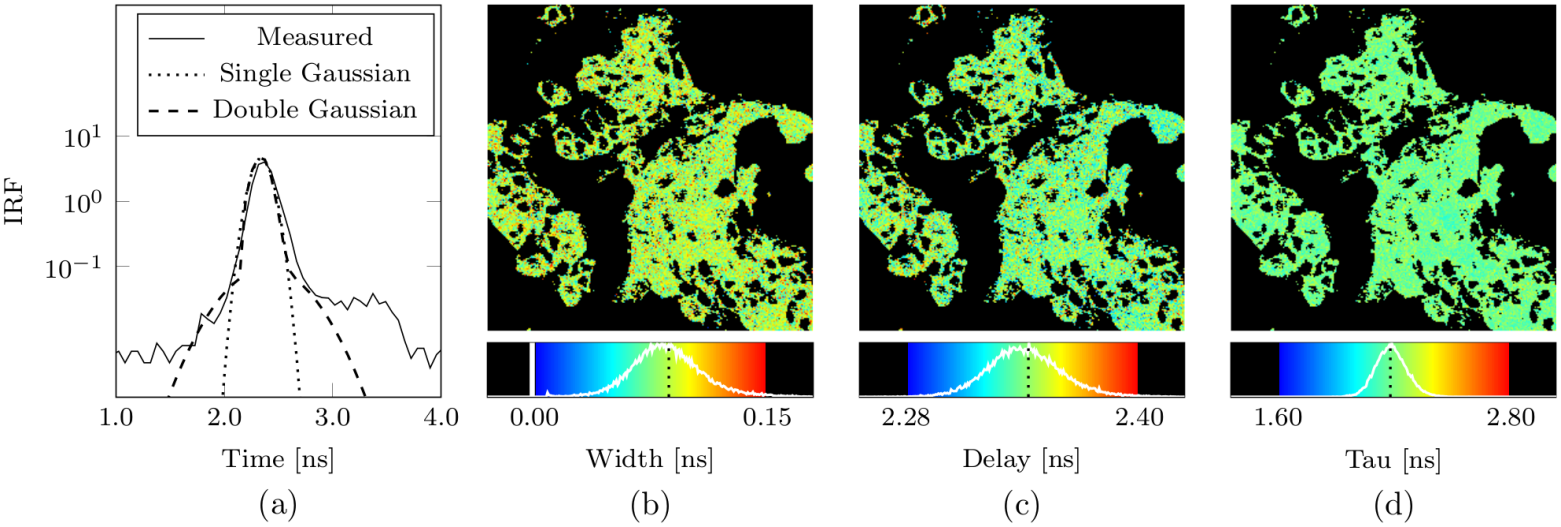
Bayesian simultaneous decay and IRF analysis. In (a) the measured and optimal single- and double-Gaussian IRF approximations as determined on application of the Bayesian SID algorithm to a single data set having over 10^7^ counts obtained by binning the data from all image pixels from a single image of the human carcinoma cell data (see Appendix A3 Human Epithelial Carcinoma Cell Preparation). In (b) and (c), the width and delay parameter estimates obtained on independent analysis of each pixel of the same human carcinoma cell data image using the Bayesian SID algorithm, assuming a mono-exponential decay and a single-Gaussian approximation, for pixels having intensities between about 350 counts and 3500 counts; in (d) the corresponding lifetime estimates. In (b), (c), and (d), the corresponding optimal single-Gaussian IRF approximation estimates are indicated in the histograms by a dotted black line.
